# Biocontrol potential and mechanism of an endophytic*Bacillus subtilis*strain KS1 against fire blight

**DOI:** 10.1128/spectrum.00240-26

**Published:** 2026-05-29

**Authors:** Jingbo Dang, Yunneng Wei, Yuan Jiang, Man Zhang, Jie Wei, Xiuli Yang, Jie Xing, Haijun Peng, Zhe Wang, Li Sun

**Affiliations:** 1 College of Life Sciences, Shihezi University 70586, Shihezi, China; 2 Xinjiang Production and Construction Corps Key Laboratory of Oasis Town and Mountain-basin System Ecology, Shihezi University 70586, Shihezi, Xinjiang, China; 3 Key Laboratory of Xinjiang Phytomedicine Resource Utilization, Ministry of Education, Shihezi University 70586, Shihezi, Xinjiang, China; 4 Agricultural Scientific Institute of 2nd Division of Xinjiang Production and Construction Corps, Tiemenguan, Xinjiang, China; Connecticut Agricultural Experiment Station, New Haven, Connecticut, USA

**Keywords:** *Erwinia amylovora*, *Bacillus subtilis*, biological control, antagonistic activity, LC–MS

## Abstract

Fire blight, caused by *Erwinia amylovora,* is one of the serious challenges in the production of pears and apples. In this study, we isolated and analyzed the biocontrol efficiency and underlying mechanisms of strain KS1, an antagonistic bacterium of *E. amylovora* obtained from the “Huoba” pear cultivar of *Pyrus pyrifolia* Nakai. Strain KS1 was identified as *Bacillus subtilis* based on multi-locus phylogenetic analysis using 16S rRNA and *rpoB* gene sequences, together with average nucleotide identity (ANI) analysis of the KS1 genome against representative *Bacillus* species. Genomic analysis revealed that KS1 harbors multiple biosynthetic gene clusters associated with antimicrobial compound production, and surfactin and bacillibactin were detected by LC–MS analysis. Laser confocal microscopy analysis demonstrated that strain KS1 can colonize the roots, leaves, and rhizosphere soil of *Pyrus betulifolia* Bunge. KS1 significantly reduced the fire blight disease index on leaves, twigs, and immature fruits of *Pyrus sinkiangensis* Yü. In addition, KS1 promoted the growth of *P. betulifolia* Bunge seedlings. Field trials during the flowering period demonstrated that strain KS1 achieved control efficacies of 84.00% and 85.39% against fire blight on individual flowers and flower clusters, respectively. These results suggest that *B. subtilis* KS1 effectively inhibits*E. amylovora* and has strong potential as a biological control agent for fire blight. This study provides insights into the antibacterial mechanisms of KS1 and supports its future development for sustainable management of fire blight.

**IMPORTANCE:**

Fire blight, caused by *Erwinia amylovora*, is a devastating disease that threatens pear output in several countries. Currently, fire blight is controlled primarily with chemical pesticides, which are causing additional issues, such as disease resistance, environmental contamination, and pesticide residues on fruit. As a result, there is a rising emphasis on creating ecologically acceptable, safe, and effective biocontrol strategies for combating fire blight in pear farming. This study explores the discovery and mechanisms of *Bacillus subtilis* KS1, which provides a novel biocontrol resource for managing pear fire blight. Strain KS1 has efficient and ecologically friendly biocontrol capabilities through a variety of mechanisms, including the production of antimicrobial lipopeptides (e.g., surfactin and bacillibactin), competition for colonization, induction of plant resistance, and direct promotion of growth. Therefore, this work demonstrates the great potential of *B. subtilis* KS1 as an alternative to chemical pesticides in sustainable pear production.

## INTRODUCTION

Fire blight, caused by the bacterium *Erwinia amylovora*, is one of the most damaging diseases affecting pome fruit trees, particularly those in the Maloideae subfamily of the Rosaceae family (1). It affects 129 plant species from 37 genera within the Rosaceae family, including not just traditional fruits like apples, pears, and quinces, but also other species (2). The pathogen penetrates the host via natural openings or injuries to above-ground structures, such as blooms, shoots, and branches. It secretes a viscous fluid called “ooze,” which aids transmission by numerous vectors, such as insects, birds, wind, and rain (3). Since its discovery in the Hudson River Valley of New York, USA, in the 1870s, the disease has spread to over 60 countries and areas around the world, including sections of Europe, Africa, the Americas, and Asia.

Several practical strategies are employed to prevent fire blight, such as trimming infected branches and applying chemical agents like copper-based compounds and antibiotics (e.g., streptomycin, oxytetracycline, and kasugamycin) (4, 5). However, these treatments often fail to stop the progression of the disease completely (6). Furthermore, the chronic use of these agents can lead to environmental pollution and pesticide residues (7). Moreover, the overuse of antibiotics has led to the development of resistant bacterial strains, prompting many countries to restrict or prohibit the use of these compounds due to concerns about public health and the environment (8).

Biological control agents (BCAs) are becoming an increasingly common option for fire blight treatment due to their effectiveness, safety, and environmental friendliness. For example, two *Pantoea ananatis* strains (BCA3 and BCA19) have been discovered as having considerable antagonistic activity against *E. amylovora* (9). Streptomyces sp. JCK-8055, isolated from pepper roots, contains compounds known as aureothricin and thiolutin, which suppress the growth of *E. amylovora* (10). Several commercial biocontrol agents have also been developed to manage fire blight, including *Pantoea vagans* C9-1 (11), *Pseudomonas fluorescens* A506 (12), *P. vagans* E325 (13), and *Bacillus subtilis* QST713 (14). However, the actual application of these biocontrol agents in the field is difficult because of their vulnerability to environmental conditions, such as desiccation, UV radiation, and rainfall (15). Current biocontrol agents for fire blight are typically ineffective and variable, and they must be used in conjunction with antibiotics (16, 17). Therefore, more microbial strains with strong antibacterial activity still need to be found in order to expand the range of biocontrol options accessible.

The rapid advancement of whole-genome sequencing technology has enabled the identification of genes and substances involved in antibacterial activity. *Bacillus* genomes contain critical gene clusters that synthesize secondary metabolites, which are essential for controlling plant diseases. Genomic sequencing of *P. ananatis* BCA19, for example, indicated gene clusters associated with the production of *E. amylovora* inhibitors, such as siderophore, andrimid, arylpolyene, and carotenoid-type terpene (9).

The “Korla fragrant pear” (*Pyrus sinkiangensis* Yü) is one of the most widely exported pear cultivars from China. It is renowned for its sweet flavor, attractive scent, and crisp flesh. However, it is extremely sensitive to fire blight. During the growth stage, the inflorescence, young fruits, and branches are especially susceptible to fire blight disease (1). In this study, we isolated *Bacillus subtilis* strain KS1 from “Huoba” pear, a variety of *Pyrus pyrifolia* Nakai. Antagonistic experiments demonstrated that strain KS1 had strong antibacterial activity against *E. amylovora*. We further conducted *in vivo* and *in vitro* tests to assess the biocontrol effects of strain KS1 against *E. amylovora* and investigated its antibacterial mechanism through whole-genome sequencing and metabolite analysis. Finally, we evaluated the ability of strain KS1 to colonize and promote the growth of “Duli” pear (*Pyrus betulifolia* Bunge), the primary rootstock for Korla fragrant pears. Our findings provide a theoretical foundation for the development of new biocontrol agents for fire blight management.

## MATERIALS AND METHODS

### Pathogenic strain, plant materials, and culture conditions

The *Erwinia amylovora* strain C1 was isolated in Korla, Xinjiang, China, and is preserved at Shihezi University. This strain was cultured for 24 h at 28°C in a nutrient agar (NA) medium (containing 3.0 g/L beef extract, 5.0 g/L peptone, 5.0 g/L NaCl, and 15.0 g/L agar, pH 7.2–7.4) (18).

Plant material, including the immature fruits, leaves, and twigs of Korla fragrant pear, as well as the seeds of “Duli” pear, was collected in Korla, Xinjiang, China (85°67′E, 41°81′N). The “Duli” seedlings were grown in a greenhouse at Shihezi University. After being surface sterilized and vernalized in moist sand for 30 days at 4°C, the “Duli” seeds were planted in a sterile substrate (a 2:1:1 mixture of potting soil, vermiculite, and perlite). The plants were grown in a climate-controlled environment at 28°C with a 12/12 h light/dark cycle for about 6 months before being tested for colonization and defense-related enzyme activity.

The “Huoba” pear variety was provided by the Zhengzhou Fruit Research Institute of the Chinese Academy of Agricultural Sciences, and cultivated at the Pear Germplasm Resource Nursery of the Agricultural Science Research Institute of the 2nd Division, Xinjiang Production and Construction Corps in Tiemenguan, Xinjiang, China.

### Isolation and screening of antagonistic endophytic bacteria

Healthy “Huoba” pear leaves were used to isolate endophytic bacteria against *E. amylovora*. The samples were cleaned with tap water and then allowed to air dry. Following three rinses with sterile water, they were washed with 75% ethanol for 1 min and 1% NaClO for 5 min. Lastly, sterilized distilled water (SDW) was used to rinse them three times. The sterilized samples were homogenized and diluted in a gradient of 0.2 mol/L PBS buffer. Then, 200 µL of the solution was placed onto the NA plate and incubated at 28°C for 2 days (19). Individual colonies were transferred to nutrient broth (NB) for culture.

### Antagonistic activity assay by agar well diffusion method

The antagonistic activity of unknown bacterial isolates against *E. amylovora* was evaluated by the agar well diffusion method (20). In brief, a 6 mm-diameter well was punctured using a sterile cork borer on an NA plate seeded with *E. amylovora* (1.0 × 10^8^–1.0 × 10^9^CFU/mL). Bacterial isolates were cultured in NB broth medium at 210 rpm and 28°C for 24 h. Subsequently, 100 μL of the culture was added to each well, while SDW served as the negative control. Plates were incubated at 28°C for 48 h, after which the inhibition zone diameter was measured. The inhibition zone was calculated as follows: the diameter of the inhibition zone (mm) = outer diameter of the inhibition zone (mm)−diameter of the well (mm).

### Genome sequencing and identification of strain KS1

#### Morphological, physiological, and biochemical characteristics

The morphological characteristics of strain KS1 cultivated for 24 h at 28°C on NB agar plates were recorded. Gram staining was performed according to the protocol of Ait-Bahadou et al. (21) and viewed using an optical microscope. A variety of biochemical assays were performed, including starch hydrolysis, the Voges-Proskauer (VP) test, the Methyl Red (MR) test, and the nitrate reduction test. The activities of KS1 protease, cellulase, and amylase were tested on agar plates containing skim milk, sodium carboxymethyl cellulose, or starch, respectively. Siderophore production was studied using the Chrome Azurol S agar medium (22, 23). Each treatment was repeated three times.

#### Molecular biological identification

Genomic DNA of strain KS1 was extracted and purified using a whole-genome sequencing kit (Tiangen Biochemical Technology Co., Ltd., Beijing, China). The 16S rRNA gene was amplified using primers 27F (5′-AGAGTTTGATCCTGGCTCAG-3′) and 1492R (5′-GGTTACCTTGTTACGACTT-3′) (24). The *rpoB* gene was amplified with primers *rpoB*-F (5′-AGGTCAACTAGTTCAGTATGGAC-3′) and *rpoB*-R (5′-AAGAACCGTAACCGGCAACTT-3′) (24). PCR products were purified and sequenced, and the obtained sequences were compared with reference sequences in the NCBI database using BLAST.

For phylogenetic analysis, the 16S rRNA and *rpoB* gene sequences of strain KS1 and related type strains were concatenated. Multiple sequence alignments were performed using ClustalW in MEGA X (25). Phylogenetic trees were reconstructed using the Maximum Likelihood method based on the Kimura 2-parameter model (26). The robustness of the tree topology was assessed by bootstrap analysis with 1,000 replicates (27).

#### Genome sequencing and analysis

Whole-genome sequencing of strain KS1was performed by General Biol Inc. (Anhui, China). Coding sequences (CDSs), transfer RNAs (tRNAs), and ribosomal RNAs (RNAs) were predicted with GeneMarkS, tRNAscan-SE v2.0, and RNAmmer-1.2, respectively. The predicted CDSs were then aligned using BLAST against several public databases, including NR, Swiss-Prot, COGs, GO, and KEGG (28). Secondary metabolite biosynthetic gene clusters (BGCs) in strain KS1 were identified using antiSMASH 7.1.0 (https://antismash.secondarymetabolites.org/) (29). The genome sequencing data were deposited in the NCBI database under accession number PRJNA1310167.

Average nucleotide identity (ANI) analysis between strain KS1 and representative *Bacillus* type strains was performed using the Integrated Prokaryotic Genome and Pan-genome Analysis web server (https://nmdc.cn/ipga/) (30). Pairwise ANI values were used to generate a heatmap and dendrogram to assess the genomic relatedness of KS1 to other *Bacillus* species.

### *In vitro* antagonistic activity of strain KS1 against *E. amylovora*

Strain KS1 was cultured in NB medium at 28°C and 210 rpm for 24 h. The culture was centrifuged at 6,000 rpm for 10 min, and the supernatant was filtered through a 0.22 μm membrane to obtain a cell-free filtrate. The antagonistic activity of the filtrate against *E. amylovora* was evaluated using the agar well diffusion method as described above, with 80 μL of filtrate added to each well. NB liquid medium served as a negative control. Each treatment was replicated three times.

### Extraction, bioactivity assay, and molecular mass determination of KS1 antibacterial compounds

#### Extraction and antibacterial assay of antibacterial compounds of KS1

Strain KS1 was streaked onto NA media and incubated at 28°C for 24 h. To prepare the seed culture, a single colony was inoculated into 10 mL of NB medium and cultivated for 24 h at 28°C and 210 rpm shaking. The seed culture (1% vol/vol) was transferred to 200 mL of Landy fermentation medium and fermented for 36 h under identical conditions (31). After fermentation, the broth was centrifuged at 4°C for 10 min at 8,000 rpm. The supernatant was then filtered through a 0.22 μm membrane for sterilization.

The antibacterial active compounds were extracted from the sterilized cell-free supernatant by methanol extraction following acid precipitation at pH 2.0 (32). The precipitate was collected by centrifugation at 12,000 rpm for 10 min and extracted twice with methanol. The combined methanol extracts were evaporated to yield the crude extract.

To evaluate antibacterial activity, the crude extract was redissolved in methanol, and 50 μL was added to each well using the agar well diffusion method as described above. After incubation at 28°C for 24 h, the diameter of the inhibition zone was measured.

#### TLC analysis of lipopeptide extracts

The antimicrobial lipopeptide in the KS1 cell-free supernatant was identified by thin layer chromatography (TLC) analysis, with chloroform–methanol–water (65:25:4, vol/vol/vol) as the mobile phase (33). Lipopeptides were visualized by spraying with 1% ninhydrin. The Rf values were determined according to the formula: Rf = migration distance of the solute/migration distance of the solvent. Each experiment was repeated three times.

#### Identification of lipopeptide extracts by liquid chromatograph–mass spectrometer

KS1 lipopeptide extracts, obtained as described above, were dissolved in methanol and identified using liquid chromatograph–mass spectrometer (LC–MS) analysis. The cell-free supernatant was analyzed using an AB 5600 Triple TOF mass spectrometer.

Chromatographic conditions: ACQUITY UPLC HSS T3 column (1.8 µm; 2.1 × 100mm); in the positive ion (ESI+) mode, the mobile phase A was 0.1% formic acid water, and B was a mixture of ethyl formate and acetonitrile. Gradient elution was carried out by gradually decreasing the fraction of mobile phase A from 95% to 5% and increasing the proportion of mobile phase B from 5% to 95% over 30 min at a flow rate of 0.4 mL/min.

Mass spectrum conditions: the ion source is a spray electrothermal spray at 500°C. The spray voltage is 4.5 kV in the negative ion mode. The mass range is 50 to 1,600 m/z.

#### Biocontrol assays on detached plant tissues

The biocontrol efficacy of strain KS1 against *E. amylovora* was evaluated using detached leaves, twigs, and immature fruits of Korla fragrant pear. All experiments were conducted in a climate chamber under controlled conditions. Streptomycin was used as a positive control to assess the effectiveness of the treatments.

#### Plant material preparation and bacterial suspension

Healthy Korla fragrant pear tissues (leaves, 1-year-old twigs, and immature fruits 50 days after flowering) were freshly harvested and surface-sterilized with 3% NaClO for 10 min, followed by three washes with SDW. For the preparation of bacterial suspensions, strain KS1 was cultured in NB medium at 28°C and 210 rpm for 24 h, and then adjusted to 1.0 × 10⁸ CFU/mL with SDW. *E. amylovora* C1 was cultured under the same conditions and adjusted to the same concentration. Streptomycin solution was prepared at a concentration of 100 μg/mL.

The experimental design included five treatment groups for each tissue type: (i) negative control (inoculated with SDW); (ii) positive control (inoculated with *E. amylovora* C1); (iii) treatment control (inoculated with KS1 suspension only); (iv) protective treatment (treated with KS1 or streptomycin 48 h prior to *E. amylovora* inoculation); and (v) curative treatment (inoculated with *E. amylovora* 48 h prior to KS1 or streptomycin treatment).

#### Antibacterial activity on detached leaves

Surface-sterilized leaves were injected with 100 μL of the respective suspensions (SDW, *E. amylovora* C1, or KS1) into the petioles and onto the leaf surface. For protective and curative treatments, leaves were sprayed with either KS1 suspension or streptomycin solution according to the treatment schedule. All treated leaves were incubated in a climate chamber at 28°C with a 12 h light/12 h dark photoperiod. Symptom development was assessed at 5 days post-inoculation (dpi). Disease severity was assessed based on the proportion of leaf area exhibiting symptoms (34), and the foliar necrosis index was evaluated on a scale ranging from level 1 to 13. Each experiment was repeated three times, with three leaves used for each treatment in each replicate.

#### Antibacterial activity on pear twigs

To maintain tissue turgor and viability during the 10-day incubation period, both cut ends of the surface-sterilized twigs were immediately sealed with sterile paraffin wax prior to inoculation. A 6 mm-diameter wound was created at the center of each twig using a sterile hole punch.

The experimental treatments (negative control, positive control, treatment control, protective treatment, and curative treatment) were applied as described above, and 100 μL of the respective suspensions was injected into the wound.

All treated twigs were placed on sterile trays lined with moist filter paper and covered with plastic wrap to maintain high humidity, then incubated in darkness at 28°C for 10 days. Disease severity was evaluated at 10 dpi by assessing necrosis around the inoculation sites. The vascular browning index of the twigs was scored on a scale from 0 to 4 as follows: 0 = no browning; 1 = 1%–10% browning; 2 = 11%–25% browning; 3 = 26%–75% browning; 4 = 76%–100% browning (35).

#### Antibacterial activity on detached immature pear fruits

Surface-sterilized immature fruits were wounded with a sterile needle, and 100 μL of the respective suspensions (SDW, *E. amylovora* C1, or KS1) was injected into each wound site. Protective and curative treatments were applied according to the schedule described above. Six fruits were used for each treatment, and each experiment was repeated three times. Symptom development was assessed at 5 dpi. Disease severity was measured using a scale of 0 to 4 based on the diameter of necrotic patches: 0 = no necrosis; 1 = 1–5 mm; 2 = 6–10 mm; 3 = 11–20 mm; 4 = 21–30 mm (35).

The relative protective effectiveness (RPE) was calculated as follows:

RPE (%) = [(infection index of the positive control − infection index of the treatment group)/infection index of the positive control]×100% (21).

#### Field efficacy assay during the flowering stage

A field efficacy trial was conducted during the flowering stage in April 2025 in a pear orchard naturally infected with fire blight, located in Tiemenguan (85.71°E, 40.80°N), Xinjiang, China. The tested cultivar consisted of 18-year-old Korla fragrant pear trees planted at a spacing of 4 m × 5 m. Prior to application, strain *B. subtilis* KS1 was cultured in LB liquid medium at 28°C with shaking at 180 rpm for 24 h. The bacterial cells were harvested by centrifugation (6,000 × *g*, 10 min, 4°C), washed twice with sterile water, and resuspended in sterile water. The bacterial suspension was adjusted to a concentration of 1.0 × 10⁸–1.0 × 10⁹ CFU/mL based on OD₆₀₀ measurements.

For comparison, two reference treatments were included: kasugamycin (4% aqueous solution; Shaanxi Maikailuo Biotechnology Co., Ltd., China) diluted 1,000-fold according to the manufacturer’s recommendation, and a commercial *Bacillus velezensis* wettable powder (Sichuan Lier Crop Science Co., Ltd., China) dissolved in sterile water to a final concentration of 1.0 × 10⁸ CFU/mL. All solutions were freshly prepared prior to application.

The experiment was arranged in a completely randomized design. A total of 76 pear trees were selected and randomly assigned to four treatments: sterile water control (CK), KS1 treatment, commercial biocontrol strain *B. velezensis* (ZH), and kasugamycin treatment (KSM), with 19 trees per treatment. Four major branches on each tree were labeled for subsequent disease assessment.

Sprays were applied at three phenological stages: early bloom (~5%–10% open flowers; 14 April), mid-bloom (~50% bloom; 18 April), and late bloom (~70%–80% bloom; 22 April). Treatments were administered to the entire tree with a 20 L backpack electric sprayer to ensure complete coverage of plant surfaces and no runoff. During the trial, the average temperature was around 18°C, with only a little rain occurring on April 20. Disease incidence was evaluated on 28 April, 4 May, and 10 May. Symptoms of fire blight, including necrosis, wilting, and bacterial ooze, were recorded for both individual flowers and flower clusters. After the flowering period, the average disease incidence for each treatment was calculated. Disease incidence and control efficacy were calculated using the following formulas:

Flower infection rate (%) = (number of infected flowers/total number of flowers tested)×100%.

Flower cluster infection rate (%) = (number of infected clusters/total number of clusters tested)×100%.

Control efficacy (%) is [(disease incidence of the control − disease incidence of the treatment)/disease incidence of the control]×100% (36).

### Assessment of the colonization ability of strain KS1

#### Prepared for endophytic *B. subtilis* KS1-GFP

To examine the colonization ability of strain KS1, the green fluorescent protein (*GFP*) gene was fused with the pHT01 plasmid (Fenghui Biology, Changsha, China) to create the pHT01-*GFP* recombinant plasmid. The recombinant plasmid was transformed into strain KS1 by electroporation, and the positive transformants were purified. The presence of the *GFP* gene was confirmed by PCR using the following primers: forward primer (with a BamH I site), 5′-caattaaaggaggaaggatccATGAGTAAAGGAGAAGAACTTTTCACTG-3′, and reverse primer (with a Sma I site), 5′-cattaggcgggctgccccgggTTATTTGTATAGTTCATCCATGCCATG-3′. GFP expression was examined using a transilluminator at 480 nm. The growth of strain KS1-*GFP* in NB agar medium was compared to that of the wild type. Single colonies of the KS1 and KS1-*GFP* strains were selected and inoculated in NB medium to generate growth curves in triplicate. The OD_600_ value was measured every 5 h.

#### Greenhouse experiments for plants inoculated with *B. subtilis* KS1-GFP

The “Duli” pear was used as the host plant, as it is the primary rootstock for Korla fragrant pears. A pot experiment was conducted on 6-week-old “Duli” seedlings inoculated with the KS1-*GFP* strain to evaluate its *in vivo* colonization ability. The treatments included (i) non-inoculated“Duli” seedlings as the control, and (ii) “Duli” seedlings inoculated with the KS1-*GFP* strain. Each treatment was performed in triplicate.

For inoculation, 30 mL of KS1-GFP suspension (1 × 10⁸ CFU/mL) was sprayed onto the leaf surfaces. In parallel, 50 mL of the same suspension was applied to the soil of each pot via drip irrigation, with the irrigation system operated for 15 min during application.

After inoculation, all pots were randomly arranged in a greenhouse at 28°C under a 12 h light/12 h dark photoperiod. To maintain adequate soil moisture, each pot was irrigated with 50 mL of SDW every 2 days until sampling. At 4 dpi, leaves and roots were collected and rinsed with SDW. Rhizosphere soil samples were collected from the root zone (0–5 mm from the root surface). All samples were examined using a laser scanning confocal microscope (Nikon AX R, Japan) with an excitation/emission wavelength of 480/510 nm (37).

### The effect of KS1 on the induction of cellular defense enzyme activities in *E. amylovora*

To assess the defense responses of enzyme activities by KS1, 6-week-old “Duli” pear seedlings were subjected to various treatments (inoculation with KS1 suspension, *E. amylovora* C1 suspension, or a combination of KS1 suspension and *E. amylovora* C1 suspension, respectively), placed in a climate chamber at 28°C with 12 h of light and 12 h of darkness, and leaf samples were collected at 0, 2, 4, 6, and 8 days (38). The activities of defense-related enzymes, including polyphenol oxidase (PPO), peroxidase (POD), and phenylalanine ammonia-lyase (PAL), were measured using particular testing kits made for each enzyme by the Jiancheng Bioengineering Institute (Nanjing, China). To guarantee consistency and dependability, each treatment was carried out three times.

### Effect of KS1 on promoting the growth of “Duli” pear

The plant growth-promoting effects of KS1 were evaluated using the “Duli” pear variety. The seeds were vernalized for 30 days, surface-sterilized, and sown in sterile nutrient soil. After 20 days, uniformly grown seedlings were chosen for root irrigation treatment. A 50 mL of KS1 suspension (1 × 10⁸ CFU/L) was inoculated into the soil using drip irrigation, with 50 mL of SDW serving as the control. Root irrigation was carried out every seven days for a total of four administrations, with each treatment repeated three times. The seedlings were grown in a growth chamber with a light/dark photoperiod of 14/10 h at 28°C and 60% relative humidity. After 28 days, the following measurements were taken: plant height, maximum leaf area, fresh weight, dry weight, and root length (38).

### Statistical analysis

The statistical analysis was carried out with SPSS 17.0, and the data were plotted using Origin 2022.2. The LSD multiple comparison test was used to analyze variance at a *P*value < 0.05.

## RESULTS

### Isolation and identification of strain KS1 and its antagonistic effects on *E. amylovora*

**Fig 1:**
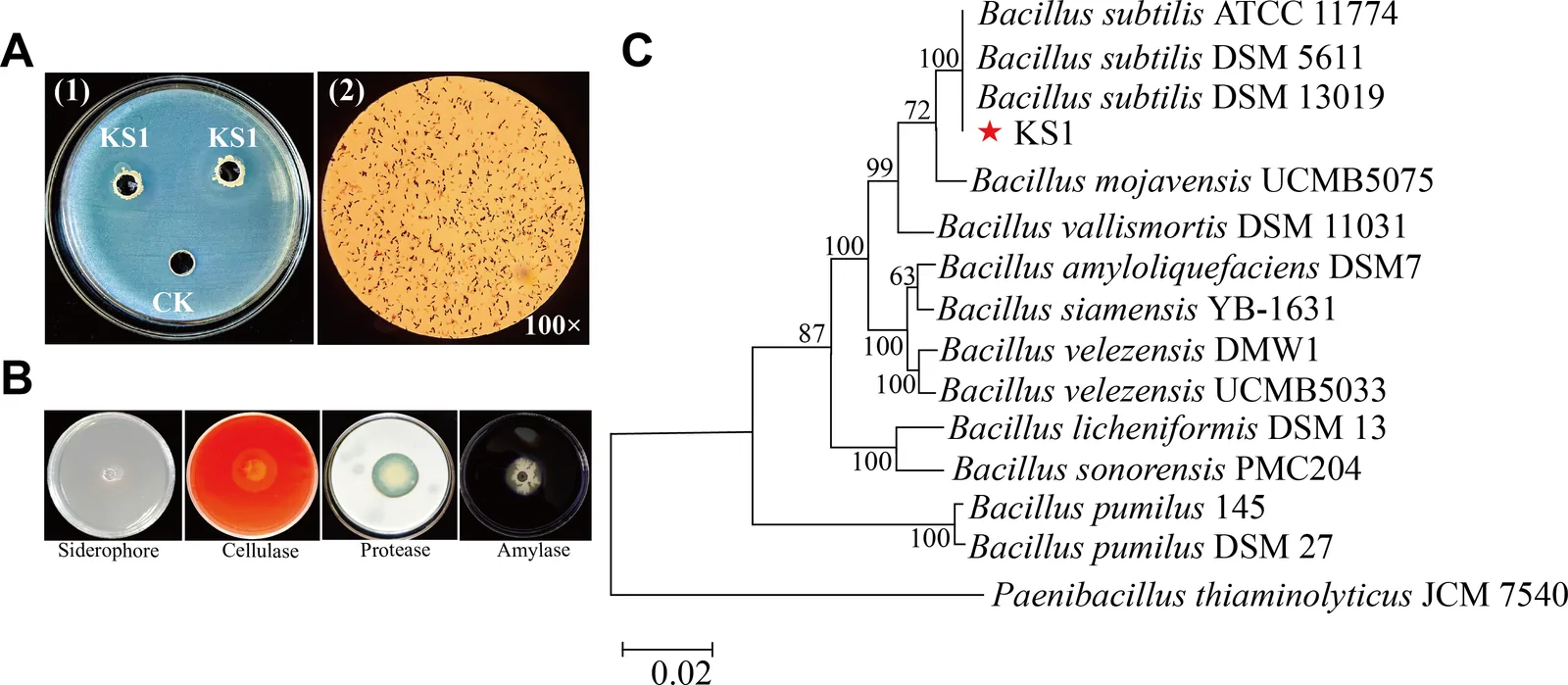
Identification and characterization of the bacterial strain KS1. (**A**)Antagonistic activity of strain KS1 against *E. amylovora* on NA plates (A1)and microscopic observation of KS1 after Gram staining (A2).(**B**)Extracellular enzyme activity assay of KS1: siderophore production, cellulase detection, protease detection, and amylase detection. (**C**)Phylogenetic tree based on concatenated 16S rRNA and *rpoB* gene sequences. The tree was reconstructed using the Maximum Likelihood method (Kimura 2-parameter model) in MEGA X with 1,000 bootstrap replicates.

Through isolation, purification, and antagonism screening, a strain designated as KS1 was obtained. It exhibited strong antagonistic activity against *E. amylovora*, with an inhibition zone diameter of21.60 ± 0.03mm(Fig. 1A1). The KS1 strain was a gram-positive rod that produced white colonies (Fig. 1A2). KS1 was positive in the starch hydrolysis test, catalase test, VP test, nitrate reduction test, and indole test, but negative in the MR test (Table 1).

**TABLE 1:** Morphological, physiological, and biochemical characteristics of strain KS1^*a*^

Test item	Strain KS1	*B. subtilis* DSM 10 (39)
Shape	Rod	Rod
Color of colony	White	White to creamy white
Motility	+	+
Gram staining	+	+
Starch hydrolysis	+	+
Voges-Proskauer (VP)	+	+
Methyl Red (MR)	−	−
Nitrate reduction test	+	+
Indole test	−	−
Catalase test	+	+

^*a*^ “+” and “−” represent positive and negative reactions, respectively.

The presence of plant growth-promoting properties of microbes associated with plant growth enhancement was investigated for strain KS1 by plate assays (40). It can create a number of hydrolytic enzymes and active substances, including cellulase, protease, amylase, and siderophores (Fig. 1B).

Phylogenetic analysis of strain KS1 was carried out using the Maximum Likelihood approach with concatenated 16S rRNA and *rpoB* sequences from KS1 and related *Bacillus* strains (accession numbers are reported inTable S1). The results showed that strain KS1 clustered with *B. subtilis* strains (ATCC 11774, DSM 5611, and DSM 13019), with strong bootstrap support (100%) (Fig. 1C). The 16S rRNA and *rpoB* gene sequences of strain KS1 have been deposited in GenBank under accession numbers PX136601 andPX229898, respectively.

Based on morphological, physiological, biochemical, and phylogenetic characteristics, strain KS1 was identified as *B. subtilis*.

### Genomic analyses of strain KS1

**Fig 2:**
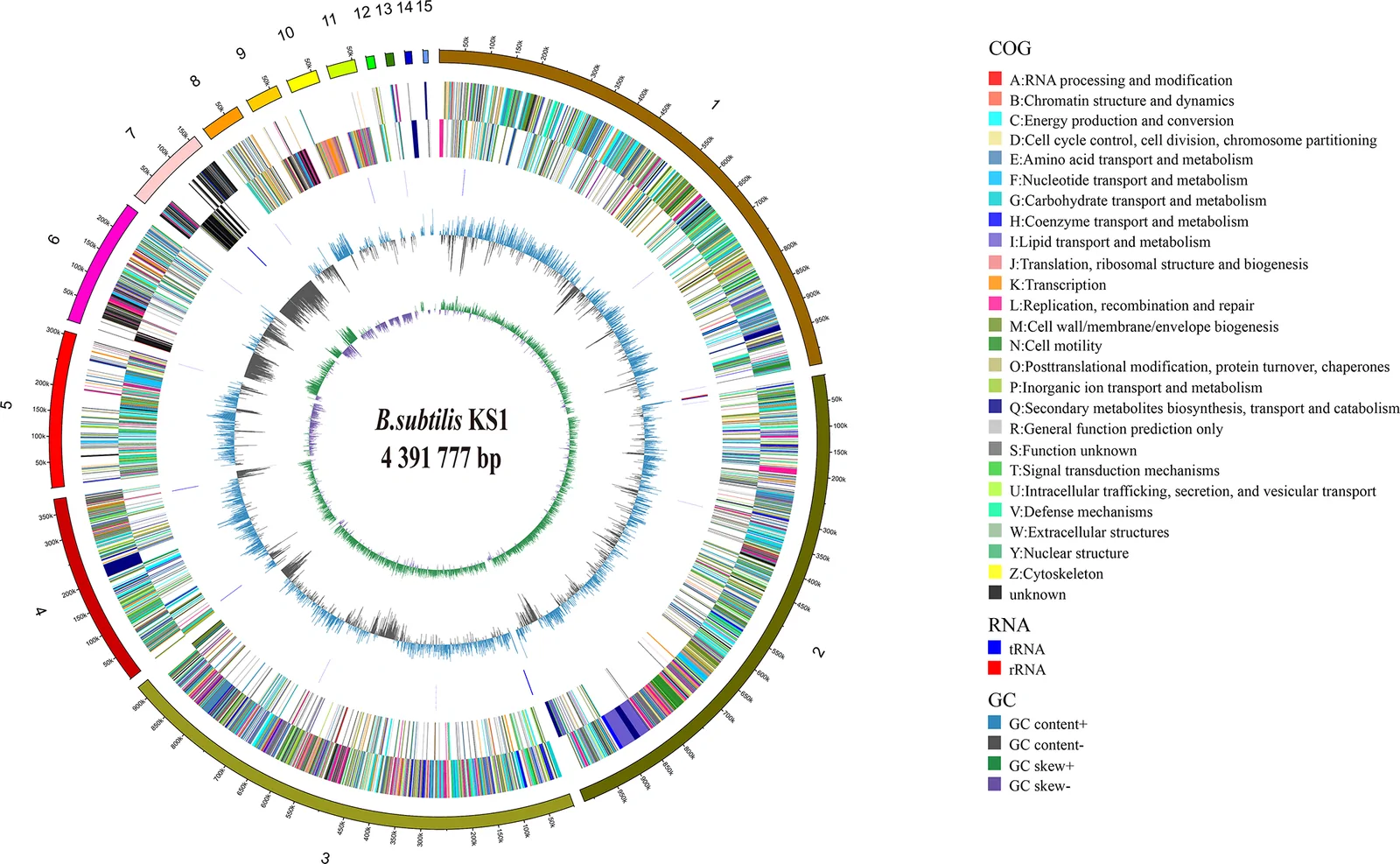
An illustration of the KS1genome. The chromosome karyotype, CDS on the positive and negative strands (various colors signifying different COG categories of the CDS), tRNA,rRNA, and GC content are listed from the outermost to the innermost circle. GC-skew is represented by the innermost circle.

The complete genome of strain KS1 consists of a 4,391,777 bp circular chromosome and no circular plasmid (Fig. 2). The G+Ccontent of the chromosome was 43.10%. A total of 4,610 CDSs were predicted, with an average length of 843.56 bp (Table S2), accounting for 88.55% of the entire genome sequence. The chromosome has 78 tRNAs and 3 rRNAs. Functional analysis revealed that 4,564, 4,101, and 4,294 of the 4,610 complete CDSs could be annotated by the NR, COG, and KEGG databases, respectively (Table S2 and Fig. S1A and B). Additionally, GO annotation enriched 9,231 genes, categorizing them into biological processes, cellular components, and molecular functions (Fig. S1C).

**Fig 3:**
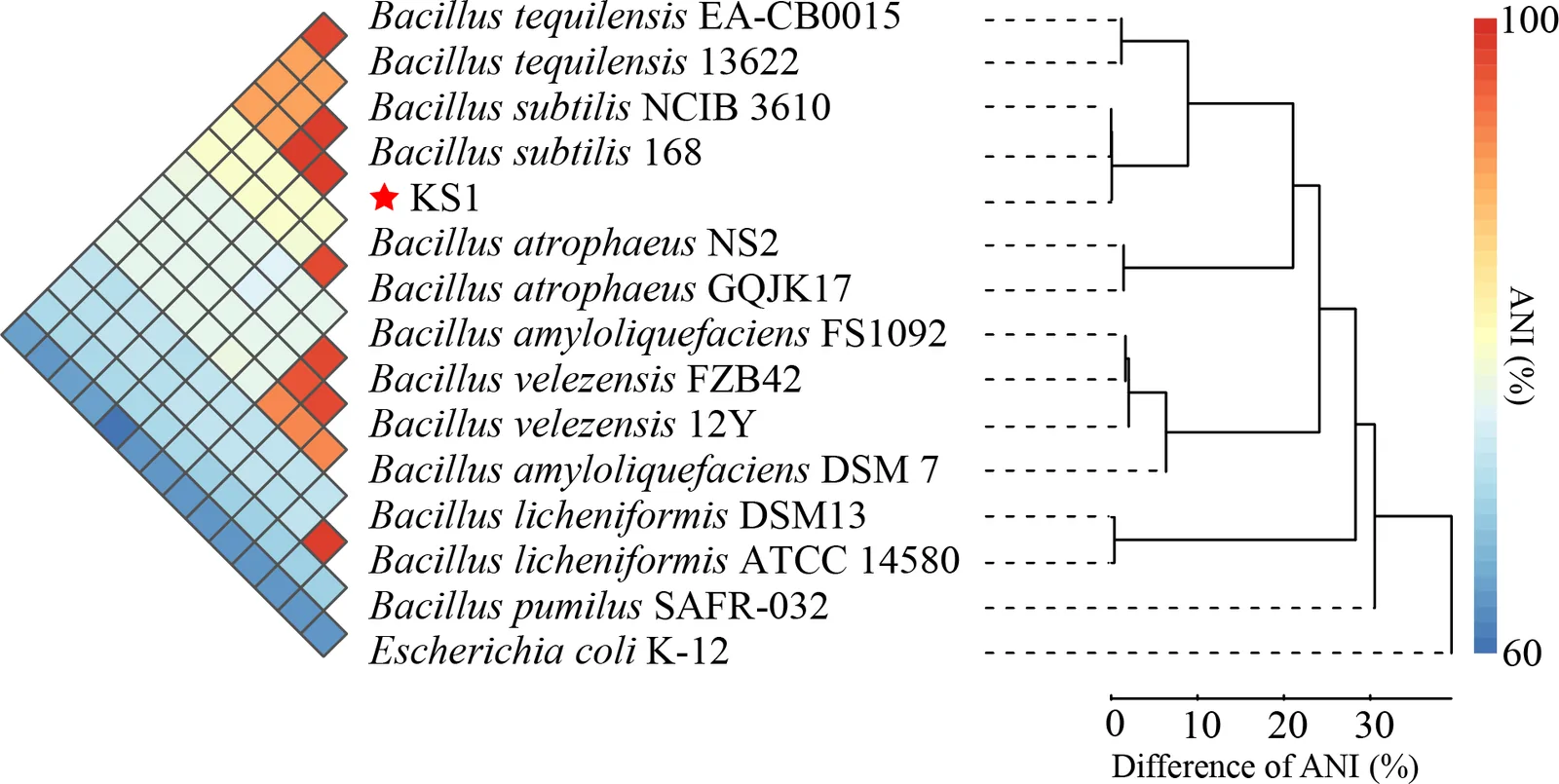
A heatmap and dendrogram of pairwise ANI values between strain KS1 and *Bacillus* type strains. The color gradient represents ANI values ranging from 60% (blue) to 100% (red).

Whole-genome ANI analysis revealed that KS1 clustered with *B. subtilis* strains, with ANI values of 99.83%–99.93% for *B. subtilis* 168 (Fig. 3). These values exceed the 95% species demarcation criteria (41), indicating that strain KS1 belongs to *B. subtilis*.

### Secondary metabolite prediction related to biocontrol function

Anti-SMASH analysis found nine BGCs in the KS1 genome that had a high degree of similarity (≥80%) to known gene clusters. The BGCs produce a wide range of secondary metabolites, including non-ribosomal peptides (NRPs) and ribosomally synthesized and post-translationally modified peptides (RiPPs). They represent the biosynthetic genes that encode for the synthetases of bacilysin, subtilosin A, pulcherriminic acid, and bacillibactin in genomic region 1; bacillaene and fengycin in genomic region 2; sublancin 168 in genomic region 3; sporulation killing factor and surfactin in genomic region 4, respectively (Table 2). According to the anti-SMASH prediction results above, the KS1 genome contains genes that encode lipopeptide antibiotics produced by NRPSs, such as surfactin and fengycin.

**TABLE 2:** Description of partial secondary metabolite BGCs in the KS1 genome^*a*^

Region	Cluster	Type	MIBIG accession	Metabolite	Similarity (%)
Region 1	3	Other	BGC0001184	Bacilysin	100
Region 1	4	RiPP: thiopeptide	BGC0000602	Subtilosin A	100
Region 1	5	Other	BGC0002103	Pulcherriminic acid	100
Region 1	6	NRPS	BGC0000309	Bacillibactin	100
Region 2	2	NRPS, polyketide	BGC0001089	Bacillaene	100
Region 2	3	NRPS	BGC0001095	Fengycin	80
Region 3	2	RiPP: lanthipeptide	BGC0000558	Sublancin 168	100
Region 4	1	RiPP: head-to-tail cyclized peptide	BGC0000601	Sporulation killing factor	100
Region 4	2	NRPS	BGC0000433	Surfactin	82

^*a*^ Genomic analysis was performed using the Antibiotics and Secondary Metabolites Analysis Shell (anti-SMASH) and the MIBiG database. The BGCs listed above refer to those gene clusters that share a gene similarity of ≥80% with the known antibiotic gene clusters in the MIBiG database. NRPS stands for non-ribosomal peptide synthetase.

**Fig 4:**
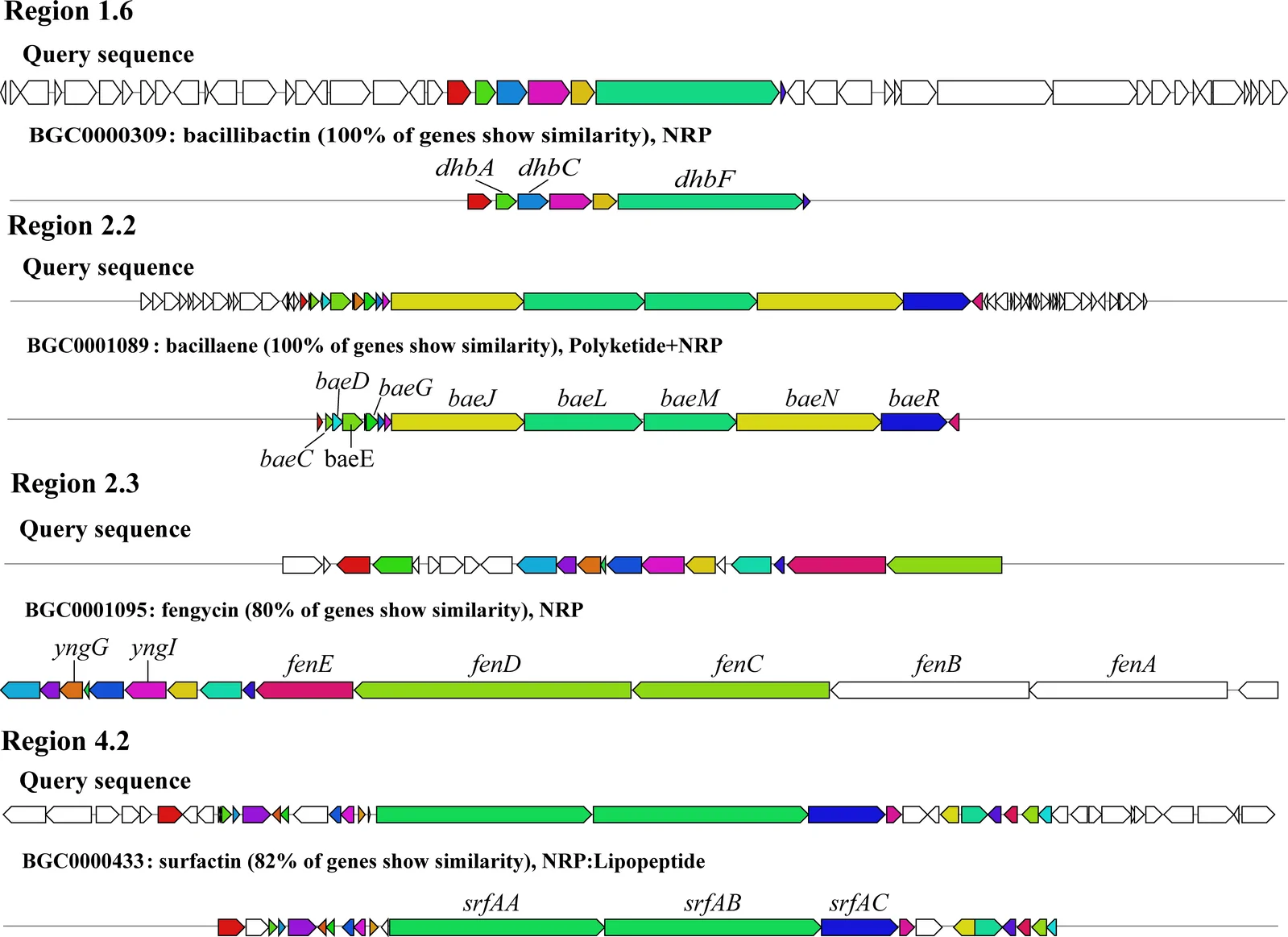
Comparative analysis of the BGCs of the query sequences in genomic regions 1, 2, and 4 of *B. subtilis* KS1 and the clustering results in the MIBiG database.

Genomic analysis was performed using antiSMASH and the MIBiG database (https://mibig.secondarymetabolites.org/), finding key biosynthetic genes, additional biosynthetic genes, regulatory genes, and other genes (Fig. 4). The bacillibactin gene cluster includes the three core genes involved in biosynthesis, such as *dhbA*, *dhbC*, and *dhbF*; the core biosynthetic genes of the bacillaene gene cluster (*baeC*, *baeD*, *baeG*, *baeJ*, *baeL*, *baeM*, *baeN*, and *baeR*). The fengycin gene cluster involved in biosynthesis (*yngG*, *yngI*, *fenA*, *fenB*, *fenC*, *fenD*, and *fenE*) was found in the KS1 genome. The KS1 genome also contains genes involved in surfactin biosynthesis (*srfAA*, *srfAB*, and *srfAC*). Based on the anti-SMASH prediction results, we discovered that the KS1 genome contains genes for lipopeptide antibiotics generated by non-ribosomal peptide synthetases (NRPS), such as fengycin, surfactin, and bacillibactin. We therefore hypothesize that the antibacterial activity of strain KS1 is connected to lipopeptide production, which will be investigated further.

### *In vitro* antibacterial activity of KS1 against *E. amylovora*

**Fig 5:**
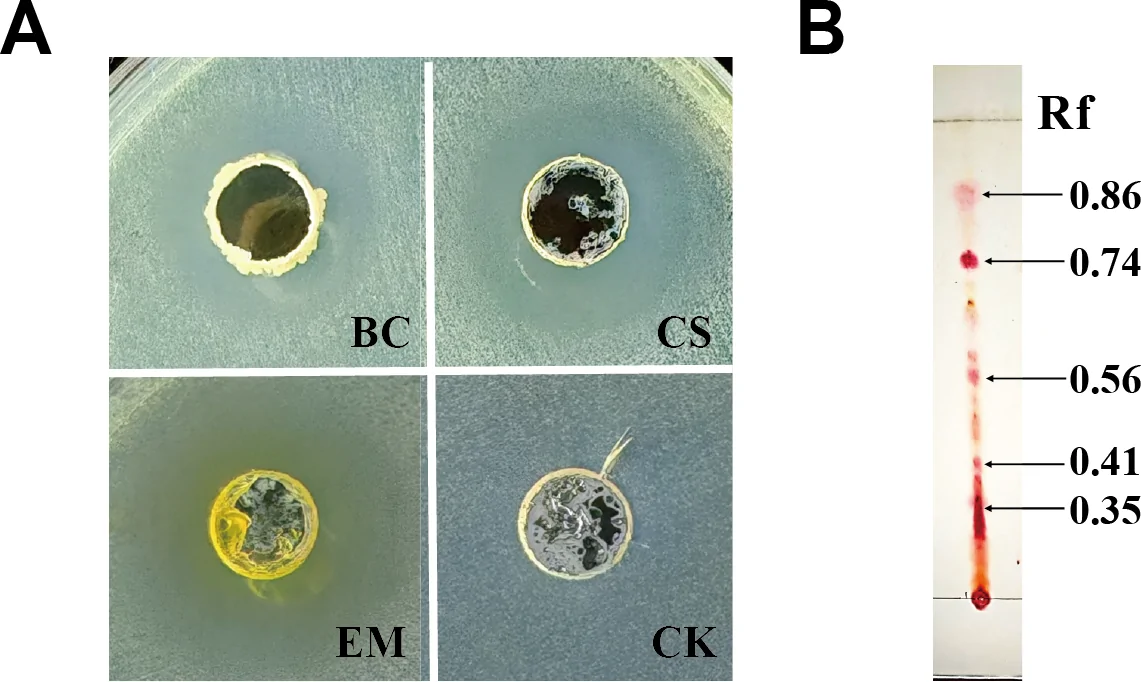
Antagonistic activity and stability analysis of antibacterial metabolites from KS1. (**A**)Antibacterial activity of different fractions from KS1 against *E. amylovora*. BC, bacterial suspension; CS, cell-free supernatant; EM, methanol extract of CS; CK, methanol control. (**B**)TLC for chemical profiling of the methanol extract components.

The antibacterial activity of KS1 against *E. amylovora* was determined using an agar well diffusion assay. The treatments included bacterial culture (BC), cell-free supernatant (CS), and methanol-extracted supernatant (EM). Methanol alone was used as the control (CK). The results showed that the EM exhibited stronger antibacterial activity, with an inhibition zone of 22.01 ± 0.24mm compared to 20.13 ± 0.12mm for CS, representing an increase of 8.8%–9.8% (Fig. 5A).

TLC analysis was performed to determine the lipopeptide profile in EM. After ninhydrin staining, various red spots were identified using a chloroform/methanol/water mobile phase, including five different spots with Rf values of 0.35, 0.41, 0.56, 0.74, and 0.86 (Fig. 5B). Based on comparisons with previously reported data (42, 43), bacillibactin D (Rf 0.35) and surfactin-like lipopeptides (Rf 0.44–0.75) were identified in strain KS1.

### Crude lipopeptide extract identification by LC–MS

**Fig 6:**
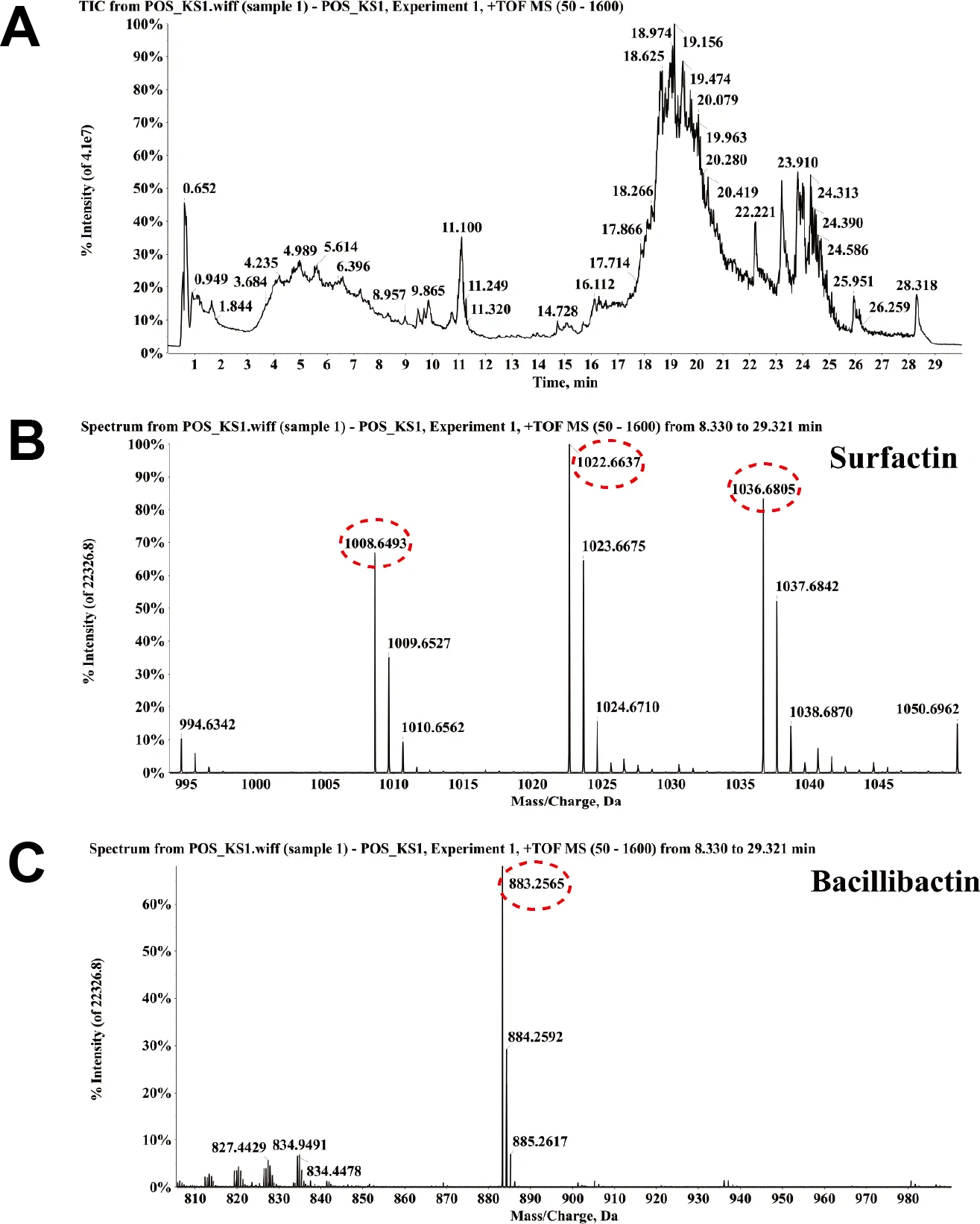
Lipopeptides identified by LC–MS in the methanol extract of the cell-free supernatant of KS1. (**A**)Total ion chromatogram (TIC) of the lipopeptides extract from KS1 in positive ion mode. (**B**)Surfactin A, B, and C mass spectra. (**C**)Bacillibactin mass spectra.

The components of the crude lipopeptide extract obtained from the methanol extract of the cell-free KS1 supernatant were examined using LC–MS (Fig. 6A). The results showed that 94 high-confidence compounds were found in the crude lipopeptide extract. Surfactins were identified based on mass spectrometry ion peaks at 1,008.6493 *m*/*z*, 1,022.6637 *m*/*z,* and 1,036.6805 *m/z* (Fig. 6B) (44). Bacillibactin was detected based on the ion peaks at 883.2565 *m/z* (Fig. 6C) (45, 46).

### Effect of strain KS1 against fire blight in detached Korla fragrant pear tissues

**Fig 7:**
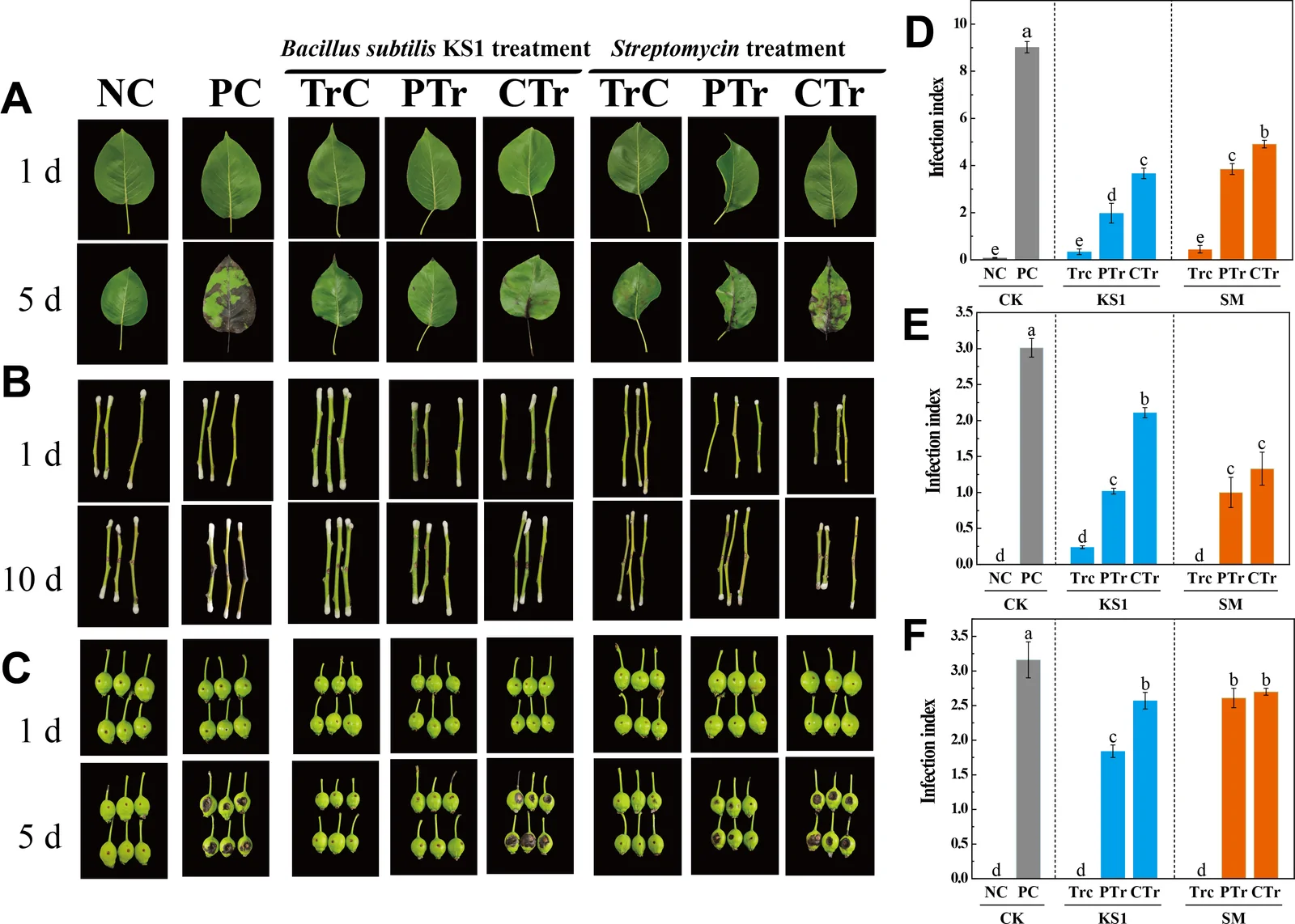
Biocontrol efficacy of KS1 against fire blight in detached Korla fragrant pear tissues. (**A and D**)Phenotypes and foliar necrosis index of leaves. (**B and E**)Phenotypes and vascular browning index of twigs. (**C and F**)Phenotypes and disease infection index of immature fruits. NC, negative control; PC, positive control; TrC, treatment control; PTr, protective treatment; CTr, curative treatment. Different letters indicate significant differences (*P* < 0.05).

In the detached pear leaf experiment, no symptoms or necrosis were observed throughout the incubation period in the negative control. By 5 dpi, large necrotic lesions had appeared on the positive control leaves, resulting in a foliar necrosis index of 9.02 ± 0.24. Both protective and curative treatments with KS1 significantly reduced the severity of fire blight compared to the positive control, with foliar necrosis indices of 1.98 ± 0.42and3.67 ± 0.22, respectively (Fig. 7A and D,Table 3). In the streptomycin control group, the foliar necrosis index for protective and curative treatments was3.85 ± 0.23and4.91 ± 0.16, respectively. These findings indicate that KS1 significantly reduces final disease progression and that protective treatment is more effective than curative treatment.

**TABLE 3:** Efficacy of *B. subtilis* KS1 on fire blight^*a*^

Treatment		Percentage of leaf necrotic spots/%	Relative control effect/%	Percentage of branch necrotic spots/%	Relative control effect/%	Diameters of lesions/mm	Relative control effect/%
Negative control		0.51±0.13g		0.00 ± 0.00g		0.00 ± 0.00f	
Positive control		49.02±0.21a		27.16±0.12a		20.67 ± 0.59a	
Treatments with KS1	TrC	1.01±0.11f		0.51 ± 0.11f		0.00 ± 0.00f	
	PTr	1.18±0.08f	78.16±5.76a	5.66 ± 0.14d	66.11 ± 0.19a	4.34 ± 0.44e	41.62 ± 2.76a
	CTr	4.27±0.19d	59.35±1.92b	9.33 ± 0.05b	29.87 ± 0.99b	14.34 ± 0.76d	18.44 ± 4.12b
Treatments with streptomycin	TrC	1.89±0.14e		0.00 ± 0.00g		0.00 ± 0.00f	
	PTr	5.86±0.23c	57.36±2.00b	4.21 ± 0.13e	67.02 ± 7.85a	16.34 ± 1.37c	17.21 ± 3.37b
	CTr	21.65±0.18b	45.58±0.46c	7.01 ± 0.19c	56.07 ± 8.12a	18.17 ± 2.31b	14.11 ± 7.76b

^*a*^ TrC, treatment control; PTr, protective treatment; CTr, curative treatment. Different letters indicate significant differences (*P*<0.05).

In the second experiment with pear twigs, the negative control showed no necrosis during the treatment. On the other hand, necrosis first emerged in the twigs inoculated with *E. amylovora* (the positive control) by 8 dpi (data not shown) and spread quickly by 10 dpi, with a vascular browning index of 3.01 ± 0.13. Protective and curative treatments with KS1 significantly reduced the vascular browning index (to 1.02 ± 0.04and2.11 ± 0.07, respectively) (Fig. 7B and E,Table 3). In the streptomycin treatment group, the vascular browning index for the protective and curative treatments was 1.00 ± 0.21and1.33 ± 0.23, respectively.

The third experiment included bioassays on immature pear fruits. The positive control inoculated with *E. amylovora* showed symptoms at 2 dpi (data not shown). At 5 dpi, the incidence of the typical fire blight symptoms reached 90%, and the infection index was 3.16 ± 0.26 (Fig. 7C and F,Table 3). There is a significant decrease between KS1 treatment and the positive control. Infection indices for preventive and curative treatments were 1.84 ± 0.09and2.57 ± 0.12, respectively. Compared to the positive control, the KS1 treatment reduced the progression of *E. amylovora*. Streptomycin treatments significantly reduced the infection index (index = 2.61 ± .14and2.70 ± 0.05, respectively) compared with the positive control. The protective treatment with KS1 was significantly (*P* < 0.05) more effective than the curative treatment. The protective treatment of KS1 was significantly more effective than the curative treatment (*P* < 0.05).

In conclusion, KS1 treatment significantly reduced the symptoms and course of *E. amylovora* disease as compared to untreated controls in *in vivo* experiments on undetached pear tissues (*P* < 0.05). Protective application was more effective than curative treatment.

### Field efficacy of KS1 against fire blight during the flowering stage

**Fig 8:**
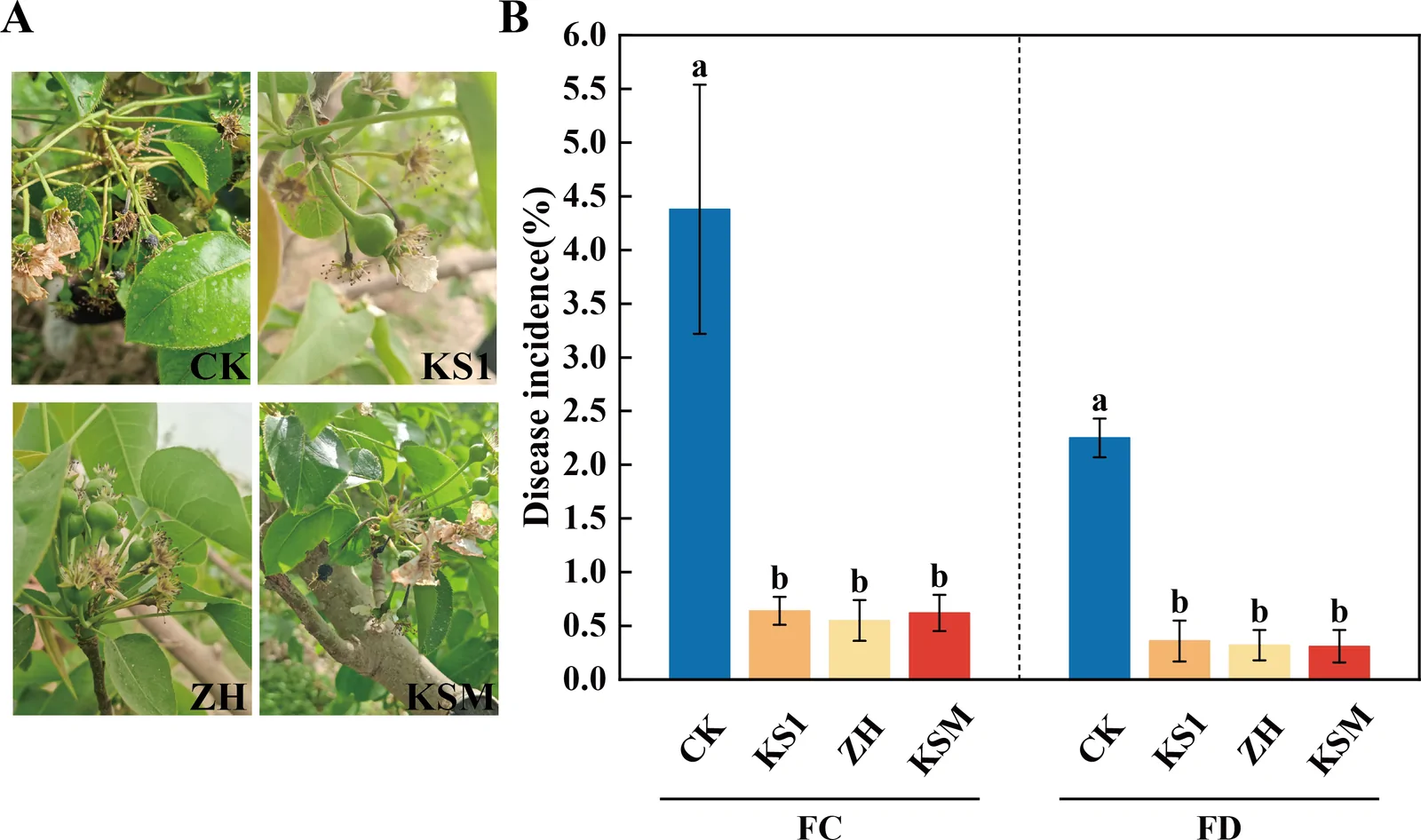
Disease incidence of flowers and flower clusters after inoculation with biocontrol strains. (**A**)Disease incidence of flower clusters and individual flowers under four treatments. (**B**)Disease incidence of flowers and flower clusters; FC,flower clusters; FD,flowers (individual flowers); CK,negative control; KS1,*B. subtilis* KS1; ZH,commercial *Bacillus velezensis* powder (Lier Crop, Sichuan); KSM,kasugamycin.Different letters indicate significant differences (*P* < 0.05).

The field efficacy of the various treatments against fire blight on flower clusters and individual flowers is presented in Fig. 8 and Table 4. Compared to the water-treated control (CK), all treatments significantly reduced disease incidence (*P* < 0.05).

**TABLE 4:** Disease incidence and control efficacy of different treatments against fire blight under field conditions^*a*^

Treatment	Disease incidence of flower clusters (%)	Control efficacy (%)	Disease incidence of flowers (%)	Control efficacy (%)
CK	4.38 ± 1.16a	–^*b*^	2.25 ± 0.18a	–
KS1	0.64 ± 0.13b	85.39 ± 4.88a	0.36 ± 0.19b	84.00 ± 8.54a
ZH	0.55 ± 0.19b	87.44 ± 5.47a	0.32 ± 0.14b	85.78 ± 6.33a
KSM	0.62 ± 0.17b	85.84 ± 5.40a	0.31 ± 0.15b	86.22 ± 6.76a

^*a*^ ZH, commercial *Bacillus velezensis* wettable powder; KSM, kasugamycin; CK, sterile water.

^*b*^ “–” not applicable.

On flower clusters, the disease incidence in the water-treated control (CK) was 4.38% ± 1.16%. All treatments significantly reduced the incidence to a range of 0.55% to 0.64%, corresponding to control efficacies of 85.39% to 87.44%. Notably, strain KS1, the commercial biocontrol strain ZH, and kasugamycin all exhibited significantly lower disease incidence compared with the CK (*P* < 0.05), with no significant differences observed among these three treatments (*P* > 0.05).

A similar trend was observed on individual flowers. The disease incidence in the CK was 2.25% ±0.18%, whereas all treatments significantly decreased the incidence to between 0.31% and 0.36%, achieving control efficacies ranging from 84.00% to 86.22%. Consistent with the flower cluster results, no significant differences in efficacy were detected among the KS1, ZH, and kasugamycin treatments (*P* > 0.05).

### Colonization of strain KS1

**Fig 9:**
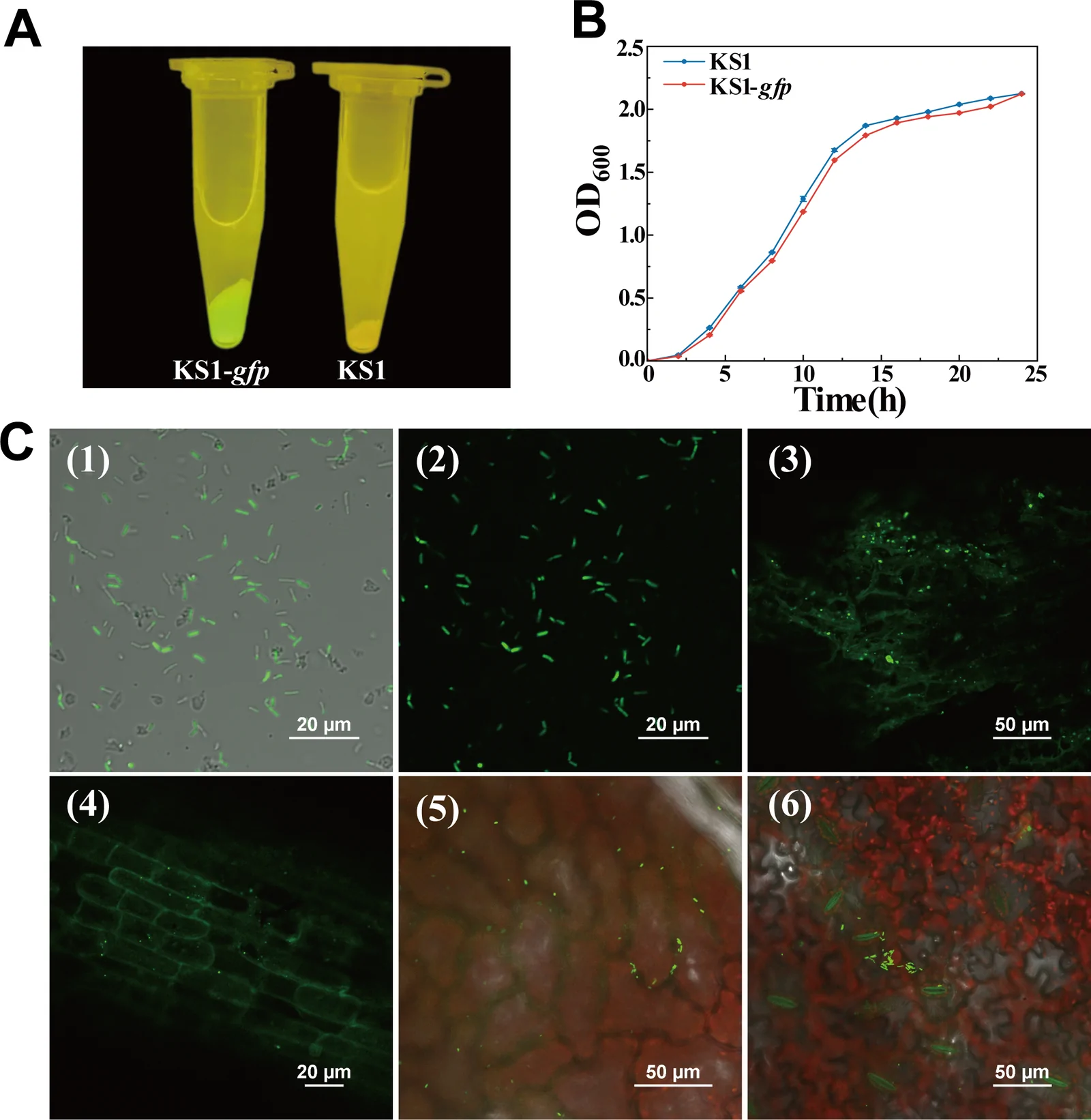
Characterization of strain KS1-*GFP* and its colonization in “Duli” pear tissues and rhizosphere soil. (**A**)Colonies of the wild-type and the KS1-*GFP* transformants under a 480 nm transilluminator. (**B**)KS1 and KS1-*GFP* growth curves on NB medium. (**C**)Confocal images showing KS1-*GFP* cells (green) and their colonization of rhizosphere soil, roots, and on the adaxial/abaxial sides of “Duli” pear leaves (1, 2). Bright-field (gray) and dark-field (black) images overlaid with GFP fluorescence (3–6). Overlay images of the rhizosphere soil (3), the roots (4), the leaf adaxial side (5), and the leaf abaxial side (6). Plant tissues are outlined in gray and auto-fluoresce in red/orange.

The pHT01-GFP plasmid was successfully inserted into the endophytic bacterium *B. subtilis* KS1. When exposed to blue light at 480 nm, the tagged strain KS1-GFP exhibited intense green fluorescence, whereas the wild-type strain KS1 did not (Fig. 9A). Furthermore, the growth rate of the tagged strain KS1-GFP in NB liquid media was consistent with that of the wildtype (Fig. 9B). These results indicate that plasmid introduction did not affect the normal growth of KS1.

Fluorescence microscopy was used to visualize GFP-labeled KS1 cells. The KS1-*GFP* transformants emit strong green fluorescence (Fig. 9C1 and 9C2), indicating effective labeling. The KS1-*GFP* cells were broadly dispersed across the 0–2 mm rhizosphere zone at 4 dpi (Fig. 9C3). Bacterial aggregates were also visible on the root surface as KS1-*GFP* colonized the root system via the rhizosphere (Fig. 9C4).

Green fluorescent cells were observed in the leaves of “Duli” pear, with many KS1-*GFP* cells located in the intercellular spaces of the mesophyll (Fig. 9C5). Furthermore, KS1-*GFP* cells were found in clusters near stomata (Fig. 9C6), indicating a possible entrance point for these cells into leaf tissues. However, additional research is required to confirm this mechanism.

### Effect of KS1 on the defense enzyme activities in *E. amylovora*

**Fig 10:**
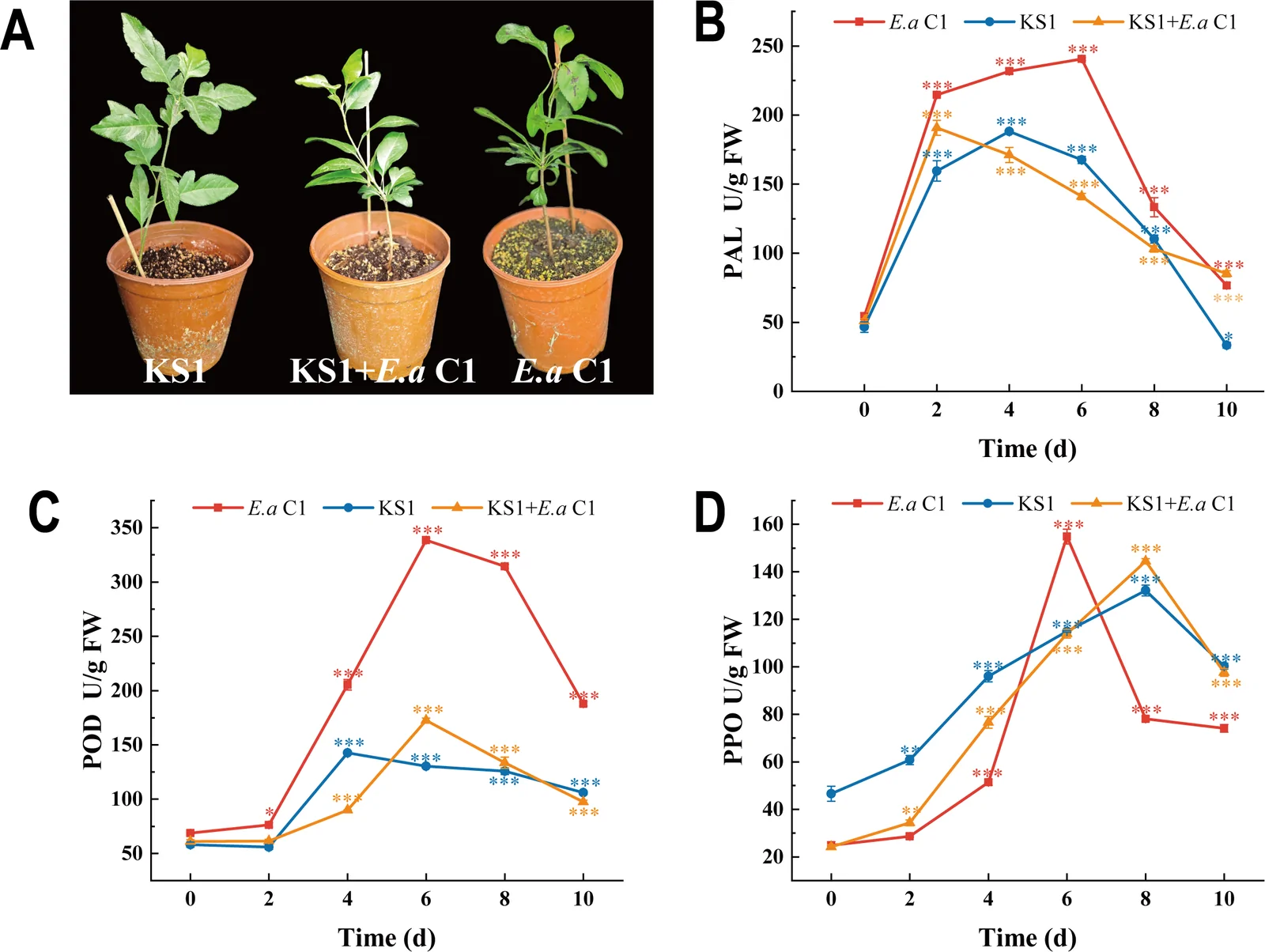
Effect of inoculating KS1 or *E. amylovora* on defense-related enzyme activities on “Duli” pear seedlings. (**A**)Phenotypes of “Duli” pear seedlings after a 4-day treatment. KS1, plants treated with KS1; KS1+E.aC1, plants treated with KS1, and then *E. amylovora* C1 24 h later; E.aC1, plants treated with *E. amylovora* C1. (**B–D**) Dynamic changes in the activities of PAL (**B**),PPO (**C**),and POD (**D**)in “Duli” pear leaves. Data are presented as the mean of three independent replicates per treatment. *P < 0.05, **P < 0.01, ***P < 0.001.

To further investigate the KS1-induced cellular defense responses, we assessed the activities of defense enzymes, including PAL, PPO, and POD, in the leaves of “Duli” pear seedlings at various time points (0, 2, 4, 6, 8, and 10 days) after treatment with KS1, *E. amylovora*, or a combination of KS1 and *E. amylovora*. Leaves treated with KS1, *E. amylovora*, or both showed considerably increased defense enzyme activity compared to day 0. Following KS1 treatment, PAL, POD, and PPO levels significantly increased after 2, 4, and 2 days, respectively, peaking at 4, 4, and 8 days (Fig. 10). These data suggest that KS1 pretreatment triggers cellular defense responses, increasing plant resistance to *E. amylovora* infection.

### *B. subtilis* KS1 promoted plant growth

**Fig 11:**
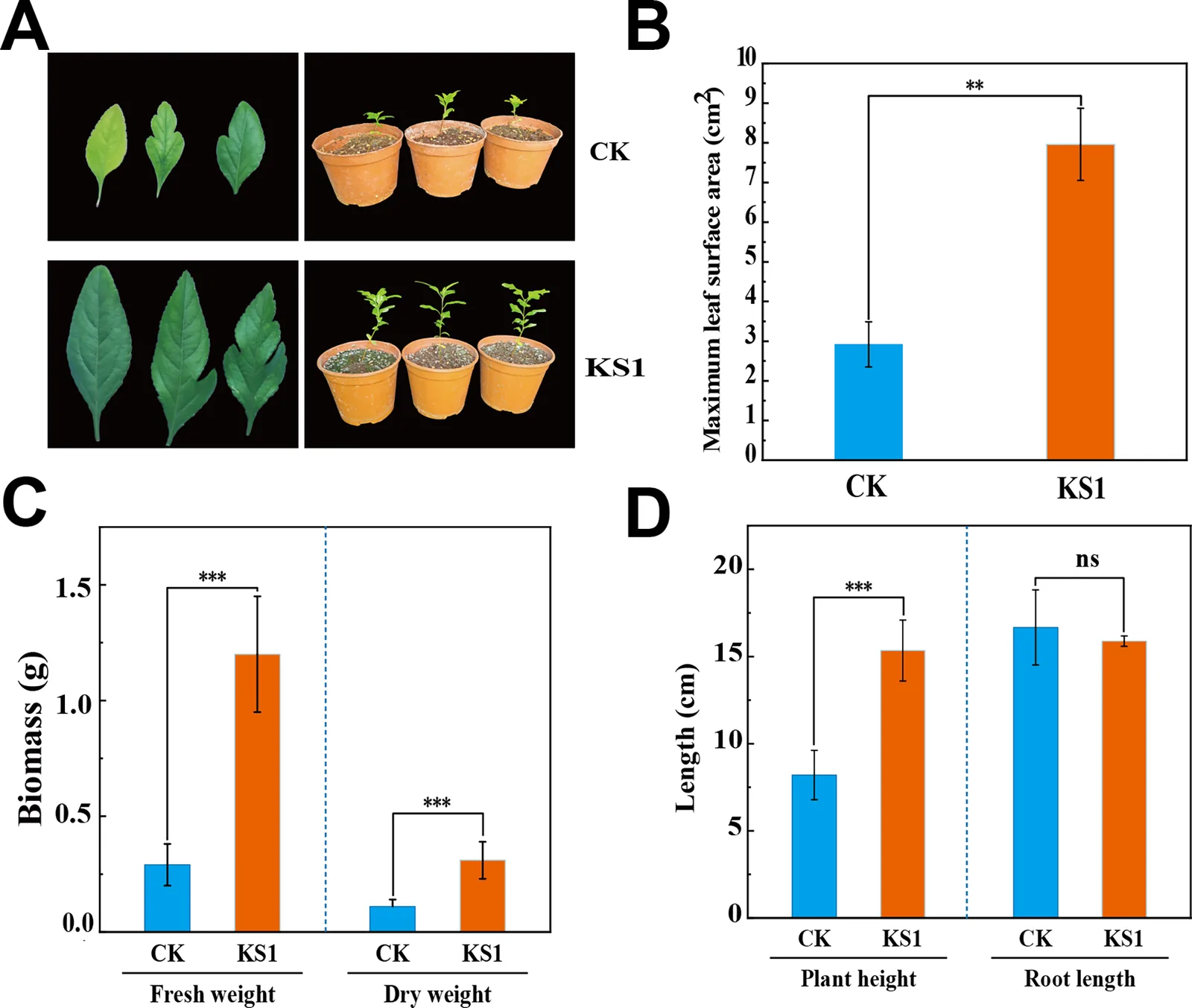
Strain KS1 promotes “Duli” pear growth after inoculation for 28 days. (**A**)Growth of “Duli” pear with and without KS1. (**B–D**) Physiological features of “Duli” pear seedlings. (**B**)Maximum leaf surface area; (**C**)biomass; (**D**)plant height and root length. CK, SDW was used as the control. *P*> 0.05, **P< 0.01, ***P< 0.001.“ns” denotes not significant, there is no statistically significant difference between the compared groups (*P* > 0.05).

Inoculation with *B. subtilis* KS1 significantly promoted the growth of “Duli” pear seedlings (Fig. 11). After 28 days of incubation, KS1-treated plants showed significantly larger maximum leaf area and stronger growth than the non-inoculated control group (Fig. 11A and B). Additionally, the KS1 treatment group had significantly greater fresh and dry weights of “Duli” pear seedlings (Fig. 11C). KS1 treatment also resulted in considerable plant height increases. There was no significant variation in root length between the KS1-treated and control plants (Fig. 11D).

## DISCUSSION

### Identification and ecological potential of the endophytic antagonist *B. subtilis* KS1

Utilizing the natural disease resistance of plants is generally considered an effective and economical strategy for disease prevention (47). However, the complexity of the polygenic resistance mechanisms in pears and the fast development of pathogens severely limit the effectiveness of resistance breeding (48). Recently, the global spread of fire blight has posed a severe threat to the production of Korla fragrant pears in Xinjiang, China. Due to the numerous negative effects of antibiotic-based chemical control, there is a growing interest in BCAs as sustainable alternatives (49, 50). While commercial BCAs like *B. subtilis* QST713 are widely used (51), identifying locally adapted strains offers distinct ecological advantages. In this study, strain KS1, an endophytic antagonist of *E. amylovora* isolated from “Huoba” pear trees, was identified as *B. subtilis*. This classification was based on a polyphasic approach, integrating 16S rRNA and *rpoB* gene analyses, whole-genome sequencing with ANI comparison, together with morphological, physiological, and biochemical characterization. The consistency among multiple independent methods significantly enhances the reliability of the taxonomic identification of strain KS1.

*Bacillus* endophytes have been confirmed to be effective against a variety of plant diseases, including pepper anthracnose, rice blast, and hemp white rot (52, 53), but they have not been widely used to control fire blight in pear. This study investigated the potential of strain KS1 as a biocontrol agent by assessing its antagonistic activity against *E. amylovora in vitro* and in pear inoculation experiments. Notably, KS1 was isolated from “Huoba” pear trees in the Korla region of Xinjiang, suggesting that it is a native, ecologically adapted endophyte. Such local adaptation may enhance its competitiveness and colonization capacity compared to non-local strains (54). These findings support the potential of *B. subtilis* KS1 as an endophytic bacterium for managing fire blight.

### Antagonistic mechanisms of *B. subtilis* KS1 against *E. amylovora*

The *Bacillus* genus controls plant diseases primarily by suppressing pathogens, competing for ecological niches, and activating plant systemic resistance (55). These bacteria use 5%–10% of their genomes to synthesize several antagonistic secondary metabolites (56). Non-ribosomal peptides, lipopeptides, polyketides, and siderophores are all important bioactive molecules. Lipopeptides, particularly the cyclic surfactins, iturins, and fengycins, are produced by NRPS complexes and have a broad spectrum of efficacy against plant pathogens (42, 57).

Genome mining of strain KS1 revealed several BGCs that produce antimicrobial compounds, such as surfactin, bacillaene, bacillibactin, and bacilysin. These metabolites are well documented to contribute to the suppression of a broad range of plant diseases (58). Interestingly, the secondary metabolite BGC profile of KS1 is highly similar to that of the commercial biocontrol strain *B. subtilis* QST713 (59), indicating a comparable biochemical capacity for broad-spectrum disease suppression.

However, KS1 was isolated as a native endophyte from pear tissues in the Korla region, which may provide significant ecological benefits, such as better host adaptability, colonization efficiency, and environmental fitness in local settings. Furthermore, its endophytic lifestyle may enhance deeper contacts with host tissues, improving persistence and biocontrol performance *in planta*. Taken together, these characteristics suggest that KS1 is a locally adapted and promising alternative to current commercial biocontrol agents for fire blight treatment.

Surfactin is an important cyclic lipopeptide that is produced by *Bacillus* species. The main antimicrobial mechanism of surfactin is disrupting pathogen cell membranes by interacting with lipid components. Furthermore, surfactin can induce cell death by damaging non-membrane targets, such as proteins and nucleic acids (57). Iron is an essential cofactor in several important biochemical processes, including electron transfer and catalysis, as well as oxygen binding in almost all species (60). *Bacillus* species generate high levels of bacillibactin through the non-ribosomal peptide pathway, competing with pathogens for Fe³^+^ in the environment. This strategy promotes their own growth while inhibiting the growth of pathogens caused by iron deficiency (61, 62). These substances are often generated by a single strain of *B. subtilis*, resulting in a higher antibacterial activity than the individual compounds (63). In this study, we found that lipopeptides extracted from the KS1 cell-free supernatant significantly inhibit the growth of *E. amylovora*. We identified the above compounds using LC–MS analysis and confirmed the existence of surfactin A, B, and C, as well as bacillibactin, in the crude lipopeptide extract of KS1. As a result, we hypothesized that surfactants and bacillibactin are the most likely antibiotics against *E. amylovora*.

As a result, the strong efficacy of KS1 could be attributed to both its ability to create antimicrobial metabolites and its potential for endophytic colonization. Compared to biocontrol strains that rely primarily on epiphytic colonization, such as *B. subtilis* QST713, KS1’s ability to colonize internal plant tissues may contribute to more stable persistence and sustained activity within the host, potentially reducing the impact of environmental variability on biocontrol performance (12, 15). However, these potential advantages must be validated by direct comparison investigations with existing commercial strains.

### KS1 enhances defense-related enzyme activities in pear seedlings

Many beneficial rhizospheric and endophytic bacteria can boost plant immunity by activating defense-related responses that may be linked to systemic resistance (64). These responses are an effective strategy for reducing reliance on chemical fungicides (65) and can be activated by a variety of biotic and abiotic stimuli, including salicylic acid (SA), jasmonic acid (JA), heat, UV light, and antagonistic microorganisms (66).

PAL is a crucial enzyme in the phenylpropanoid pathway, helping to produce defense-related chemicals like lignin and SA (67). PPO and POD are also key components of plant defense, helping to build lignin and accumulate oxidative phenolics, which strengthen cell wall integrity and limit pathogen invasion (68, 69). In this study, KS1 treatment significantly increased the activities of PAL, POD, and PPO in “Duli” pear seedlings. These results indicate that KS1 can activate plant defense-related physiological responses. However, whether this activation is due to systemic acquired resistance or induced systemic resistance remains to be determined through additional investigations of defensive signaling pathways and associated marker genes.

### KS1 is capable of colonizing plant tissues

For a biocontrol bacterium to be effective, it needs to be able to colonize the rhizosphere of the host plant and other ecological niches. For example, a mutant strain of *Pseudomonas chlororaphis* PCL1391, which has a reduced ability to colonize roots, cannot effectively control *Fusarium oxysporum* in tomato plants (70). Bacterial endophytes often begin in the soil and spread to plants via the rhizosphere and root system. Alternatively, these bacteria can enter a plant’s phyllosphere as epiphytes through wounds and fractures caused by pathogen invasion, wind, insect vectors, or natural openings like stomata and hydathodes (71). The biocontrol bacteria can colonize several parts of leaf tissues, such as the xylem vessels, palisade mesophyll cells, epidermal cell surfaces, and the intercellular gaps of the spongy mesophyll (72). In this study, KS1 colonization in “Duli” pear seedlings was confirmed. In addition to accumulating in the soil and colonizing the root surface, KS1 was able to enter leaves via foliar spray and colonize the intercellular gaps of the mesophyll. However, the invasion mechanisms of KS1 require further investigation.

### KS1 has a positive effect on plant growth

Plant growth-promoting bacteria constitute a long-term strategy for increasing crop yield while minimizing environmental damage (73). Endophytic *Bacillus* species can enhance plant development through a variety of direct and indirect methods, including hydrolytic enzyme synthesis, nutrient acquisition facilitation, and plant defense response activation (74, 75).

Strain KS1 was found to be capable of producing many hydrolytic enzymes, including cellulase, protease, and amylase, all of which are linked to increased nutrient cycling and availability in the rhizosphere (76). In addition, KS1 created siderophores, which may help the host plant get iron (77). These functional features are congruent with the observed impacts on plant performance, as the KS1 treatment dramatically increased leaf development, biomass accumulation, and overall growth in “Duli” pear seedlings.

Taken together, these data imply that KS1 incorporates a variety of favorable features, such as growth stimulation, antimicrobial action, and endophytic colonization, which may all contribute to better plant health. This multimodal profile demonstrates KS1’s potential as a suitable choice for long-term management of pear production systems.

### Conclusions

In this study, *B. subtilis* KS1 was isolated from the “Huoba” pear and shown to be highly effective against *E. amylovora*. KS1 produces a variety of bioactive lipopeptides, including surfactins and bacillibactin, which may have an important antagonistic role in *E. amylovora*. KS1 was found to significantly reduce the fire blight disease index in the leaves, twigs, and immature fruits of Korla fragrant pear trees, while also promoting growth and biomass accumulation in “Duli” pear seedlings. Furthermore, field trials during flowering showed that the control efficacy of KS1 was comparable to that of the commercial biocontrol strain *Bacillus velezensis* ZH and the antibiotic kasugamycin, with all treatments significantly outperforming the control and no significant differences observed among them. Therefore, *B. subtilis* KS1 is a promising microbial resource with disease-suppressive and growth-promoting potential for managing pear fire blight.

## Data Availability

The GenBank accession numbers for the 16S rRNA gene and whole-genome sequences of strain KS1 are PX136601 and PRJNA1310167, respectively.

## SUPPLEMENTAL MATERIAL

Fig. S1 spectrum.00240-26-s0001.tif Genome annotation, metabolic pathways, and predicted secondary metabolites of *B. subtilis* KS1.

Fig. S2 spectrum.00240-26-s0002.tif Construction of the recombinant vector pHT01-GFP and verification of the KS1-GFP strain.

Tables S1 and S2, Fig. S1 and S2 spectrum.00240-26-s0003.docx Table S1: GenBank accession numbers of 16S rDNA and rpoB gene sequences for strain KS1 and related *Bacillus* type strains used in phylogenetic analysis. Table S2: Genomic features of KS1. Fig. S1: Genome annotation, metabolic pathways, and predicted secondary metabolites of *B. subtilis* KS1. Fig. S2: Construction of the recombinant vector pHT01-GFP and verification of the KS1-GFP strain.

ASM does not own the copyrights to Supplemental Material that may be linked to, or accessed through, an article. The authors have granted ASM a non-exclusive, world-wide license to publish the Supplemental Material files. Please contact the corresponding author directly for reuse.
