# Supplementary figures and images for: Biocontrol potential and mechanism of an endophyticBacillus subtilisstrain KS1 against fire blight
Source: Microbiol Spectr. 2026 May 29;14(7):e00240-26. doi: 10.1128/spectrum.00240-26 (PMC13340183; doi:10.1128/spectrum.00240-26)

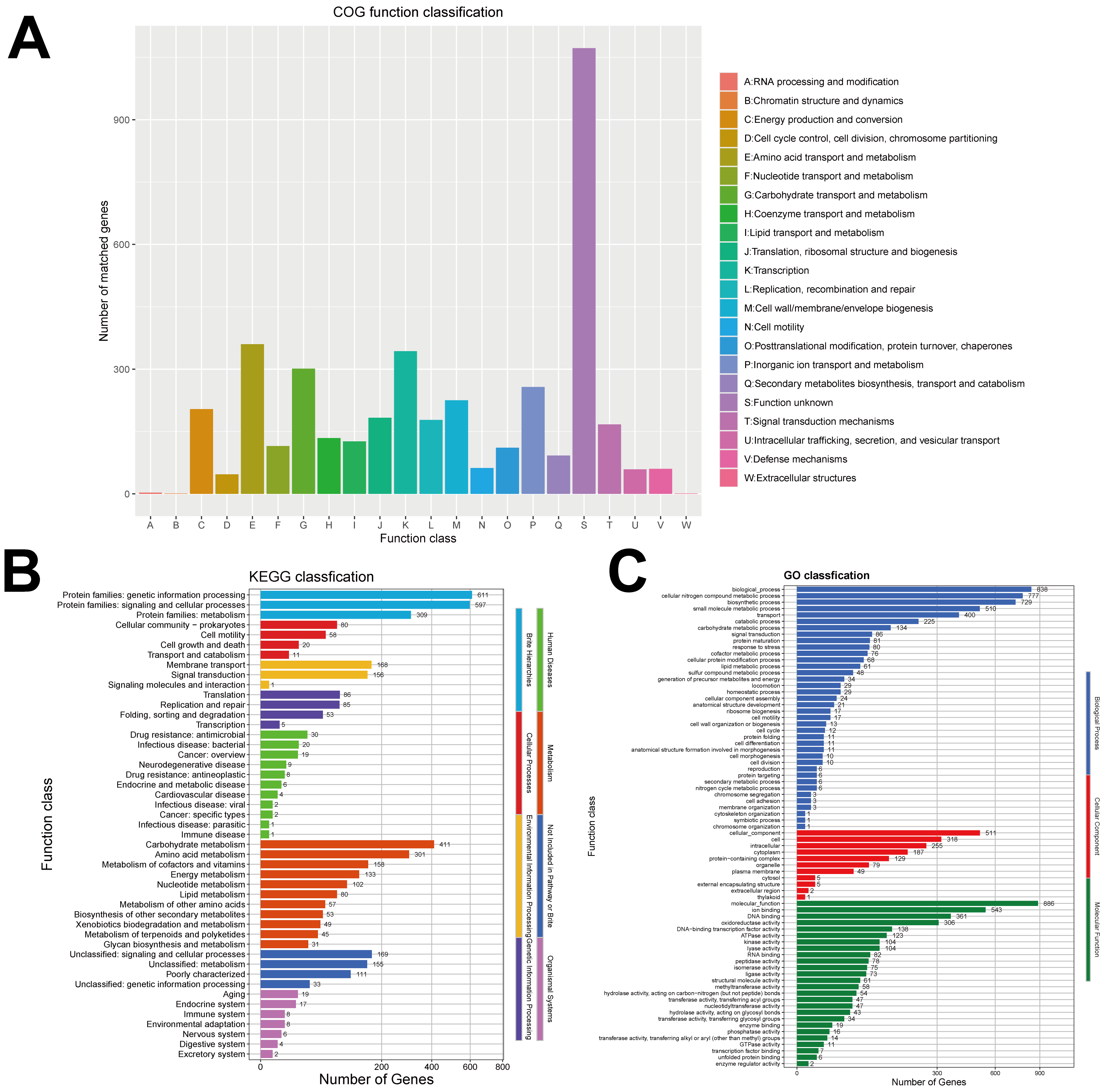

Supplement: Fig. S1 — Genome annotation, metabolic pathways, and predicted secondary metabolites of B. subtilis KS1. [file spectrum.00240-26-s0001.tif]

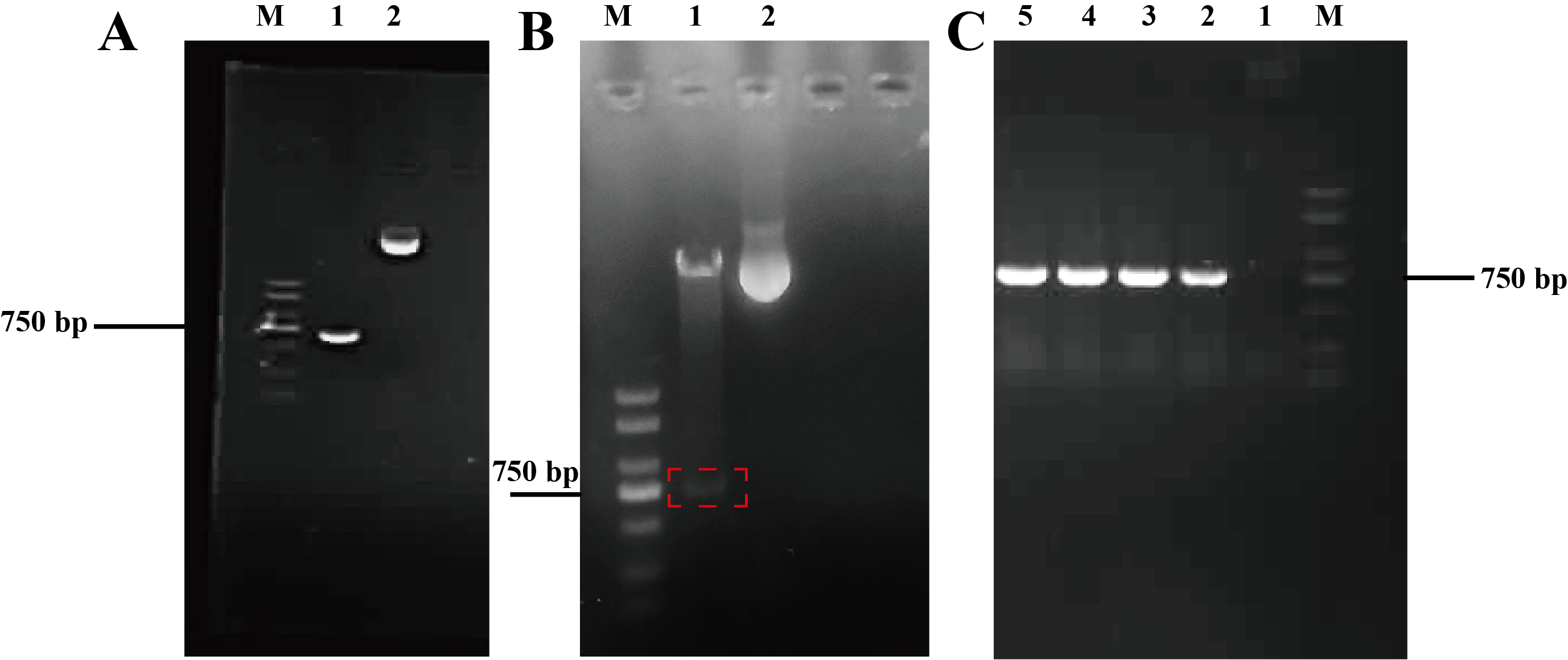

Supplement: Fig. S2 — Construction of the recombinant vector pHT01-GFP and verification of the KS1-GFP strain. [file spectrum.00240-26-s0002.tif]
