# Supplementary material for: Biocontrol potential and mechanism of an endophyticBacillus subtilisstrain KS1 against fire blight
Source: Microbiol Spectr. 2026 May 29;14(7):e00240-26. doi: 10.1128/spectrum.00240-26 (PMC13340183; doi:10.1128/spectrum.00240-26)
Supplement: Tables S1 and S2, Fig. S1 and S2 — Table S1: GenBank accession numbers of 16S rDNA and rpoB gene sequences for strain KS1 and related Bacillus type strains used in phylogenetic analysis. Table S2: Genomic features of KS1. Fig. S1: Genome annotation, metabolic pathways, and predicted secondary metabolites of B. subtilis KS1. Fig. S2: Construction of the recombinant vector pHT01-GFP and verification of the KS1-GFP strain. [file spectrum.00240-26-s0003.docx]

**Biocontrol potential and mechanism of an endophytic *Bacillus subtilis* strain KS1 against fire blight**

Jingbo Dang^a,^¹, Man Zhang^a,^¹, Yuan Jiangᵇ, Yunneng Wei^a^, Jie Weiᵇ, Xiuli Yang^a^, Jie Xing^a^, Haijun Peng^a^, Zhe Wang^a^, Li Sun^a,^*

(a College of Life Sciences, Shihezi University, Shihezi 832003; Xinjiang Production and Construction Corps Key Laboratory of Oasis Town and Mountain-basin System Ecology; Key Laboratory of Xinjiang Phytomedicine Resource Utilization,Ministry of Education.

b Agricultural Science Research Institute of the 2nd Division, Xinjiang Production and Construction Corps, Tiemenguan 841005, Xinjiang)

1 These two authors contributed equally to this work.

* Corresponding author: Li Sun

Table S1. GenBank accession numbers of 16S rDNA and *rpoB* gene sequences for strain KS1 and related *Bacillus* type strains used in phylogenetic analysis.

| **Species** | 16S rDNA | *rpoB* |
| --- | --- | --- |
| KS1 | PX136601 | PX229898 |
| *Bacillus amyloliquefaciens* DSM7 | NR_118950.1 | FN597644.1 |
| *Bacillus licheniformis* DSM 13 | NC_006270.3 | NC_006270.3 |
| *Bacillus mojavensis* UCMB5075 | CP051464.1 | CP051464.1 |
| *Bacillus pumilus* 145 | CP027116.1 | CP027116.1 |
| *Bacillus pumilus* DSM 27 | CP046130.1 | CP046130.1 |
| *Bacillus siamensis* YB-1631 | NZ_CP110268.1 | NZ_CP110268.1 |
| *Bacillus sonorensis* PMC204 | NZ_CP139190.1 | NZ_CP139190.1 |
| *Bacillus subtilis* ATCC 11774 | CP026010.1 | CP026010.1 |
| *Bacillus subtilis* DSM 5611 | CP120603.2 | CP120603.2 |
| *Bacillus subtilis* DSM 13019 | NZ_CP120621.2 | NZ_CP120621.2 |
| *Bacillus vallismortis* DSM 11031 | NZ_CP026362.1 | NZ_CP026362.1 |
| *Bacillus velezensis* DMW1 | NZ_CP114180.1 | NZ_CP114180.1 |
| *Bacillus velezensis* UCMB5033 | NC_022075.1 | NC_022075.1 |
| *Paenibacillus thiaminolyticus* JCM 7540 | NZ_AP043707.1 | NZ_AP043707.1 |

Table S2. Genomic features of KS1.

| **Feature** | **Chromosome Characteristics** |
| --- | --- |
| Genome size (bp) | 4 391 777 bp |
| GC Content (%) | 43.10% |
| CDS No. | 4 610 |
| tRNA No. | 78 |
| rRNA No. | 3 |
| Gene average length | 843.56 bp |
| Gene density genes per kb | 1.050 |
| Gene assigned to NR | 4 564 |
| Gene assigned to GO | 9 231 |
| Gene assigned to eggNOG | 4 101 |
| Gene assigned to KEGG | 4 294 |
| Gene assigned to Swiss-Prot | 4 197 |
| Gene assigned to CAZy | 173 |
| Gene assigned to CARD | 57 |


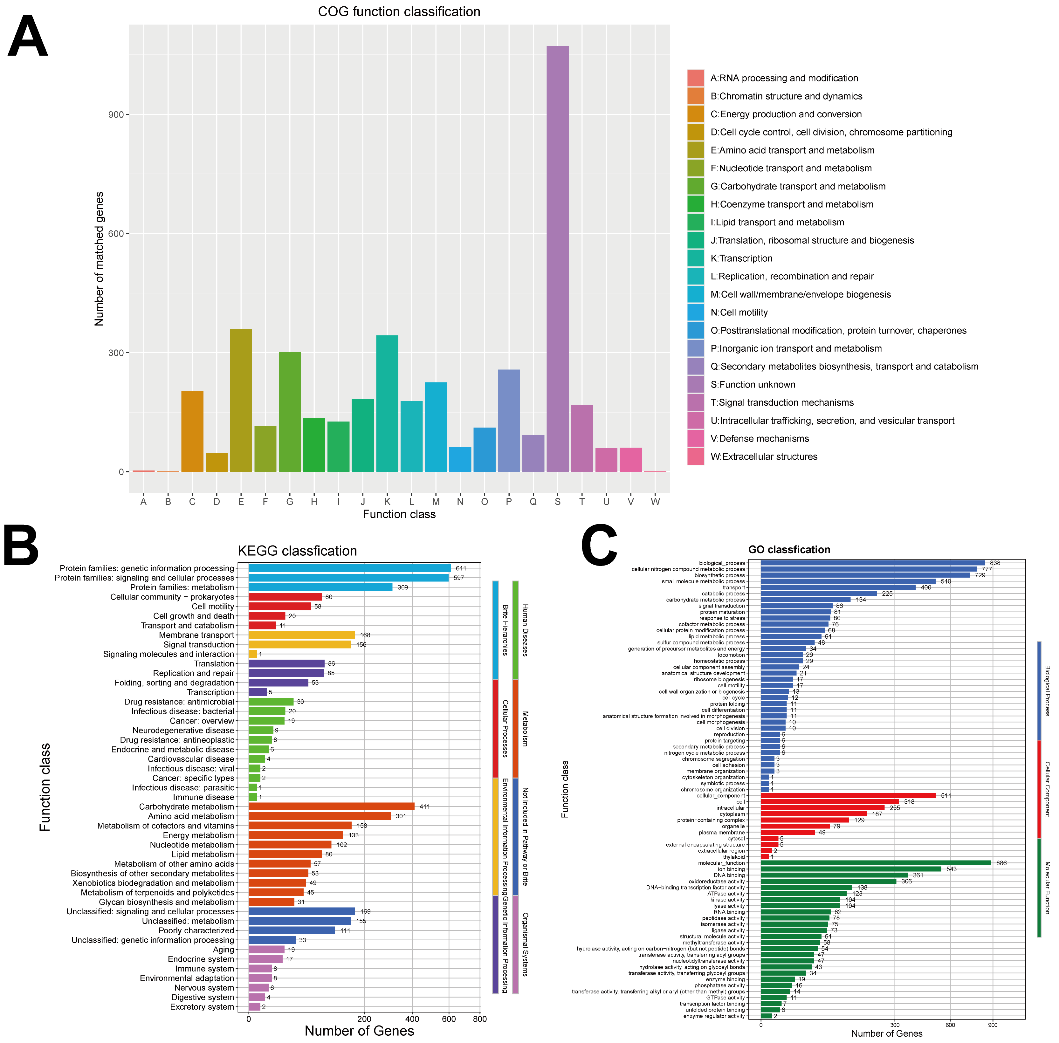


**Fig. S1** Genome annotation, metabolic pathways and predicted secondary metabolites of *B. subtilis* KS1. (A) KS1 genome annotation by COG. (B) KS1 genome annotation by KEGG pathway. (C) KS1 genome annotation by GO.


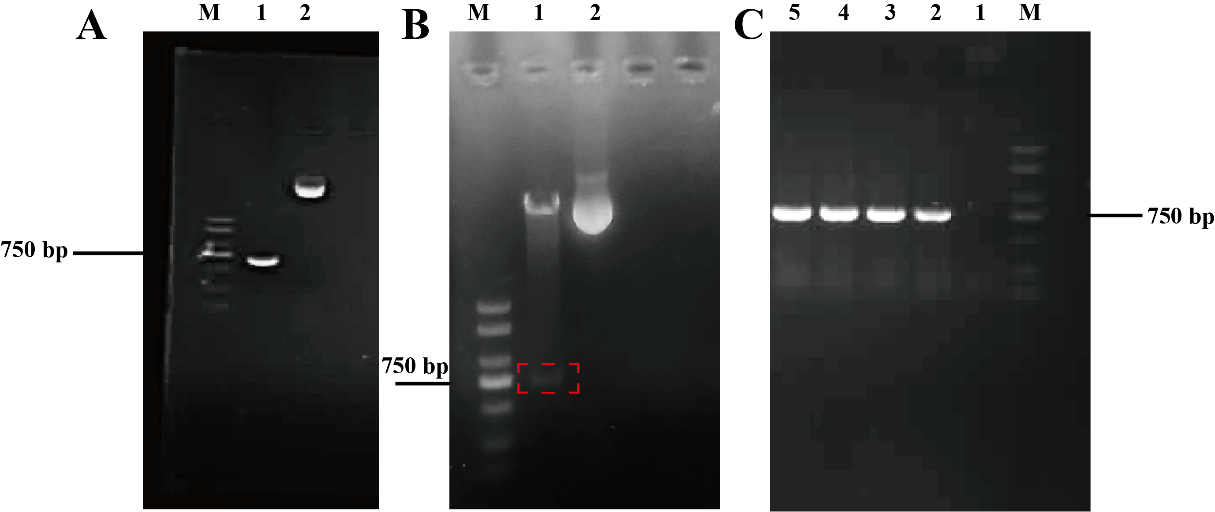


**Fig. S2** Construction of the recombinant vector pHT01-GFP and verification of the KS1-GFP strain. (A) BamH I and Sma I digestion of pHT01 vector and the GFP gene PCR product. Line 1: GFP gene following double digestion. Line 2: pHT01 vector following double digestion. (B) BamH I and Sma I digestion confirm the recombinant plasmid pHT01-GFP. Line 1: pHT01-GFP. Line 2: pHT01 vector as a control. (C) PCR verification of the recombinant strain KS1-GFP. Line 1: PCR of the wild-type strain KS1 (negative control). Lines 1–5: colony PCR detection of the recombinant strain KS1-GFP. M: DL2000 Plus DNA Marker.
